# Genome-wide association analyses of invasive pneumococcal isolates identify a missense bacterial mutation associated with meningitis

**DOI:** 10.1038/s41467-018-07997-y

**Published:** 2019-01-14

**Authors:** Yuan Li, Benjamin J. Metcalf, Sopio Chochua, Zhongya Li, Hollis Walker, Theresa Tran, Paulina A. Hawkins, Ryan Gierke, Tamara Pilishvili, Lesley McGee, Bernard W. Beall

**Affiliations:** Respiratory Diseases Branch, Division of Bacterial Diseases, National Center for Immunization and Respiratory Diseases, Centers for Disease Control and Prevention, U.S. Department of Health and Human Services, Atlanta, 30333 Georgia USA

## Abstract

Bacterial mutations predisposing pneumococcus to causing meningitis, a more severe form of invasive pneumococcal disease (IPD), are largely unknown. Knowledge of such mutations may improve our understanding of pathogenesis and inform preventive strategies. Here we report a pneumococcal *pbp1b* gene mutation (*pbp1b*A641C causing N214T change in PBP1b transglycosylase domain) that is associated with meningitis in an exploratory cohort of IPD patients (n = 2054, p = 6.8 × 10^−6^), in an independent confirmatory cohort (n = 2518, p = 2.3 × 10^−6^), and in a combined analysis (n = 4572, p = 3.0 × 10^−10^). Patients infected by the *pbp1b641C* genotype pneumococci show 2.8-fold odds (95% CI 1.7 to 4.8) of meningitis compared to those infected by non*-pbp1b641C* pneumococci, after controlling for pneumococcal serotype, antibiotic resistance, and patient age. The *pbp1b*A641C change results in longer time needed for bacterial killing by antibiotic treatment and shows evidence of being under positive selection. Thus, a pneumococcal mutation conferring increased antibiotic tolerance is associated with meningitis among IPD patients.

## Abstract (web summary)

Meningitis is a severe form of invasive pneumococcal disease (IPD). To study the contribution of bacterial genomic variation, here Li et al. perform whole genome sequencing of pneumococcal isolates from IPD patients and identify an association for higher risk of meningitis with a *pbp1bA641C* variant

## Introduction

The Gram-positive bacterium *Streptococcus pneumoniae* (pneumococcus) frequently colonizes the human nasopharynx and can invade normally sterile body sites to cause invasive pneumococcal disease (IPD), including bacteremia, bacteremic pneumonia, and meningitis^1^. Despite effective vaccines^2,3^, IPD remains a leading cause of morbidity and mortality worldwide^4,5^. Pneumococcal meningitis is the more severe form of IPD, associated with high risk of death and permanent neurological sequelae^6,7^. Patients with suspected acute bacterial meningitis require immediate antimicrobial treatment and hospitalization^8^. Although it has long been hypothesized that pathogen–host interaction affects disease type and severity, whether specific pneumococcal genomic variations predispose the pathogen to causing meningitis over non-meningitis IPD in humans is largely unknown. Knowledge of such bacterial variations may improve the understanding of pathogenesis, inform patient management, and direct preventive strategies.

Host-pathogen interaction underlying pneumococcal pathogenesis has been studied intensively using tissue cultures and animal models^9–12^. Multiple pneumococcal virulence factors, including choline-binding protein A (CbpA)^11^, neuraminidase A (NanA)^13^, pneumococcal adherence and virulence factor A (PavA)^14^, the extracellular redox repair system^15^, and pneumolysin^16^, among others^17^, are implicated in brain endothelial cell invasion and meningeal inflammation. The significance of these gene knockouts in differentiating meningitis from non-meningitis IPD patients is less clear because these are often core genes carried by nearly all clinical isolates, yet only a minority of them cause meningitis. A comparative genomic analysis of 140 clinical isolates suggested that gene presence/absence alone was unlikely to account for the virulence behavior of pneumococci that infect human meninges^18^. Besides gene presence/absence, sequence variations such as single-nucleotide polymorphisms (SNPs) causing amino acid changes could also facilitate the adaptation of the pneumococcus to different host anatomical locations, and can be evaluated through genome-wide association study (GWAS). This type of studies has been shown to be feasible for pneumococcal populations, in which frequent recombination events reduce linkage disequilibrium^18–20^. A large, diverse sample is critical to capture the extensive genomic diversity and to detect candidate SNPs with sufficient power.

Here, we analyze whole-genome sequencing and accompanying epidemiological data of 4572 IPD isolates obtained through the Active Bacterial Core surveillance (ABCs)^21,22^. ABCs is an active, population- and laboratory-based surveillance system conducted in ten sites, representing ~34 million residents in ten U.S. states^23^. For each case of IPD, a case report containing epidemiological information and the pneumococcal isolate from a normally sterile site (available most of the time) are sent to the Centers for Disease Control and Prevention (CDC). Routine audits of the reporting clinical laboratories are performed at least once a year to ensure that all cases of disease under surveillance are being captured^23^. In this study, we include an exploratory sample (*n* = 2054) and a confirmatory cohort (*n* = 2518). The exploratory sample is a selection of invasive pneumococcal isolates in ABCs from 1999 to 2013, while the confirmatory cohort is a population-based collection of all invasive pneumococcal isolates in ABCs in 2015. Using the relatively large sample and high-quality epidemiological data, we identify a pneumococcal *pbp1b* gene mutation (*pbp1b*A641C) associated with meningitis among IPD patients. We also quantify the mutation’s effect sizes, document the current allele distribution, and characterize bacterial phenotypic changes caused by the mutation.

## Results

### Exploratory screening

The exploratory sample isolates were selected to broadly represent the pneumococcal genomic diversity in the surveillance areas from 1999 to 2013 (Supplementary Data 1). A review of case reports found that 139 (6.8%) invasive isolates were from meningitis patients and 1915 (93.2%) invasive isolates from non-meningitis patients (Table 1). Patients aged < 2 and 5–17 years with IPD had a higher frequency of meningitis compared with other age groups (Table 1). The seven serotypes included in the seven-valent pneumococcal-conjugated vaccine (PCV7) and the additional six serotypes included in PCV13 (PCV13 minus PCV7) had a slightly lower frequency of meningitis cases compared with the non-PCV13 serotype group (Table 1, odds ratio = 0.8 and 0.57 for PCV7 and PCV13 minus PCV7, respectively). No association of meningitis cases with either sample year or penicillin (PEN) susceptibility was observed (Table 1).

**Table 1:** Characteristics of invasive pneumococcal isolates in the exploratory sample

	Non-meningitis (*n* = 1915)	Meningitis (*n* = 139)	*P*-value^a^
Patient age (years)			<0.001
<2	774 (40%)^b^	74 (53%)	
2–4	535 (28%)	21 (15%)	
5–17	108 (6%)	17 (12%)	
18–64	288 (15%)	22 (16%)	
> 64	210 (11%)	5 (4%)	
Sample year			0.27
1995–1999	557 (29%)	42 (30%)	
2000–2009	868 (45%)	54 (39%)	
2011–2013	490 (26%)	43 (31%)	
Serotype group			0.034
Non-PCV13^c^	766 (40%)	69 (50%)	
PCV7^d^	527 (28%)	38 (27%)	
PCV13 minus PCV7	622 (32%)	32 (23%)	
PEN MIC (µg ml^−1^)			0.22
≤0.06	1273 (66%)	100 (72%)	
≥0.12	642 (34%)	39 (28%)	

^a^Fisher’s exact test on even distribution of meningitis isolates among each level of the indicated factor

^b^Data are number (%). Some percentages do not total 100 because of rounding

^c^PCV13 serotypes are 1, 3, 4, 5, 6A, 6B, 7F, 9V, 14, 18C, 19A, 19F, and 23F

^d^PCV7 serotypes are 4, 6B, 9V, 14, 18C, 19F, and 23F

**Fig. 1:**
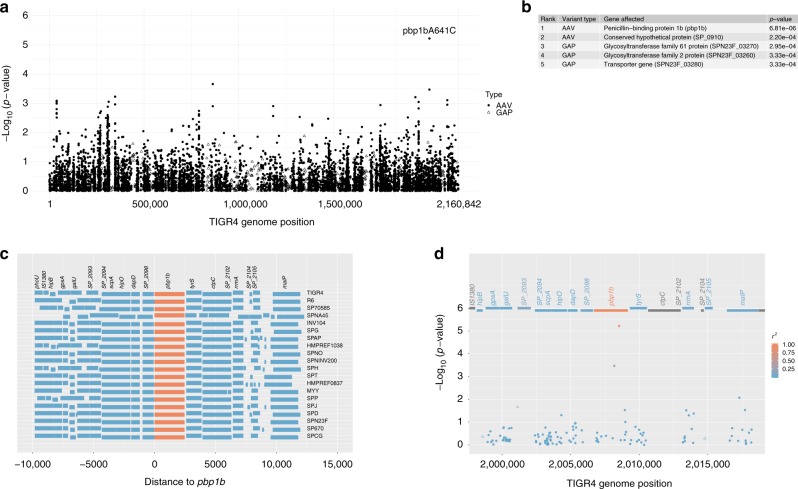
Association between pneumococcal variants and meningitis in an exploratory sample. **a** *P*-values (−log10 transformed) of the test variants assessed by a linear mixed-effects model (LMM) controlling for population structure. Coding DNA sequence (CDS) variants were mapped to the corresponding position of the TIGR4 reference genome. Solid dots: non-synonymous CDS SNPs causing amino acid variations (AAVs). Open triangles: gene absence/presence variations (GAPs). **b** A list of top five hits form the association study. **c** Genomic structure within −10 and + 10 kb of the *pbp1*b gene in 20 reference pneumococcal genomes. The *pbp1*b gene is colored in orange. **d** Regional plot of variants near the *pbp1b* gene. AAVs (solid dots) and GAPs (open triangles) are colored according to their linkage disequilibrium (r^2^ value) with the lead variant (*pbp1b*A641C)

Whole-genome sequencing and de novo assembly of the exploratory sample isolates identified 7480 synonymous coding DNA sequence (CDS) SNPs, 10,584 non-synonymous CDS SNPs causing amino acid variations (AAVs), and 1885 gene absence/presence variations (GAPs) (Table 2). All AAVs and GAPs (*n* = 12469) were assessed for association with meningitis using a linear mixed-effects model (LMM) to control for population structure, which was based on a relatedness matrix of all 18,064 CDS SNPs (Fig. 1a). Patient age, a known risk factor for meningitis^6^, was included in the LMM as a covariate. Model fitting using the FaST-LMM software identified one AAV showing the lowest *P*-value as a candidate variant (*P* = 6.8 × 10^–6^, Fig. 1b). The *P*-value was marginally significant after Bonferroni correction (adjusted *P* = 0.08). The second lowest *P*-value (*P* = 2.2 × 10^–4^, Fig. 1b) was 31-fold larger than that of the candidate variant and was not significant after Bonferroni correction (adjusted *P* > 0.99). Model fitting using the GEMMA software showed similar results (Supplementary Table 18). The candidate variant (*pbp1b*641C) corresponded to an A to C substitution at position 641 of the reference *pbp1b* sequence (SP_2099), resulting in an asparagine (N) to threonine (T) alteration in the transglycosylase domain of the penicillin-binding protein (PBP) 1B. In the exploratory sample, 16.5% (23) meningitis patients and 6.4% (122) non-meningitis patients were infected by the *pbp1b*641C genotype pneumococci. Among the 1909 non-*pbp1b*641C isolates, 98% (1876) had sequence A at the position *pbp1b*641 while 2% (33) had a missing value.

**Table 2:** Analysis of invasive pneumococcal isolates in the confirmatory cohort

	Non-meningitis (*n* = 2340)	Meningitis (*n* = 178)	Effects	Mixed-effects logistic regression OR (95% CI)^b^	*P*-value^b^
*pbp1b*641C			Fixed		
No	2093 (89%)^a^	136 (76%)		Reference	
Yes	247 (11%)	42 (24%)		2.83 (1.65–4.84)	<0.001
Patient age (years)			Fixed		
<2	90 (4%)	18 (10%)		1.97 (1.10–3.38)	0.016
2–4	47 (2%)	2 (1%)		0.34 (0.05–1.18)	0.149
5–17	53 (2%)	7 (4%)		1.40 (0.62–3.19)	0.421
18–64	1205 (51%)	110 (62%)		Reference	
>64	941 (40%)	41 (23%)		0.46 (0.32–0.66)	<0.001
Unknown	4 (0.2%)	0 (0%)		NA^c^	
Serotype			Random		
44 levels					
Susceptibility to six β-lactam antibiotics 19 levels			Random		

^a^Data are number (%). Some percentages do not total 100 because of rounding

^b^Mixed-effects logistic regression model with binary outcome (meningitis vs. non-meningitis) and the indicated explanatory variables

^c^The four patients with unknown age were excluded from the regression analysis

The *pbp1b* gene was conserved among 20 pneumococcal reference genomes, consistently flanked by a hypothetical protein gene (SP_2098) and the tyrosyl-tRNA synthetase gene (*tyrS*) (Fig. 1c). Variants at adjacent loci showed no significant association with meningitis and a low level of linkage disequilibrium with the candidate variant (Fig. 1d).

### Confirmatory study

**Fig. 2:**
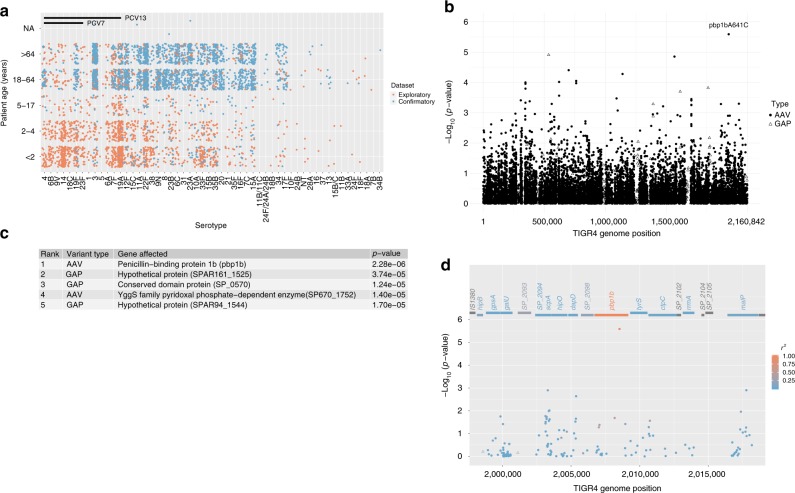
Association between pneumococcal variants and meningitis in the confirmatory cohort. **a** Comparison of patient age and serotype distribution between the exploratory study isolates (orange) and the confirmatory study isolates (blue). PCV7 and PCV13 indicate serotypes included in the pneumococcal conjugate vaccine 7 and 13, respectively. **b** *P*-values (−log_10_ transformed) of test variants assessed by a linear mixed-effects model (LMM) controlling for population structure. Coding DNA sequence (CDS) variants were mapped to the corresponding position of the TIGR4 reference genome. Solid dots: non-synonymous CDS SNPs causing amino acid variations (AAVs). Open triangles: gene absence/presence variations (GAPs). **c** A list of top five hits form the association study. **d** Regional plot of variant p-values near the *pbp1b* gene. AAVs (solid dots) and GAPs (open triangles) are colored according to their linkage disequilibrium (r^2^ value) with the lead variant (*pbp1b*A641C)

The confirmatory cohort consisted of all available invasive isolates (*n* = 2518) from the ABCs IPD cases between January 1 and December 31st 2015, representing 85.6% of all IPD cases in the ten surveillance sites (Supplementary Data 2). The proportion of meningitis isolates (7.1%) was similar to that of the exploratory sample. The majority of the confirmatory cohort patients were adults (≥ 18 years old) and were infected by non-PCV serotype pneumococci (Fig. 2a). The age and serotype distribution demonstrated the current IPD epidemiology in this population (Fig. 2a) and differed substantially from what was observed in the exploratory sample (Fig. 2a), thus allowing the confirmatory cohort to serve as an independent replication.

The primary objective of the confirmatory study was to assess whether *pbp1b*641C is associated with higher likelihood of meningitis syndrome among IPD patients. Secondary objectives included (1) to identify additional candidate variants, (2) to quantify the increased risk of meningitis associated with *pbp1b*641C in the different subgroups, and (3) to characterize the distribution of *pbp1b641C* genotype in the population.

A power analysis based on LMM estimates from the exploratory sample indicated that a sample size of that dataset (*n* = 2054) would achieve powers of 0.99, 0.97, and 0.73 in detecting association between *pbp1b641C* and meningitis at significance levels of 0.05, 0.01, and 10^−4^, respectively. The power analysis supported a sufficient sample size of the confirmatory cohort for the primary study objective.

Whole-genome sequencing and de novo assembly of the confirmatory cohort isolates identified 16,047 synonymous CDS SNPs, 17,636 non-synonymous CDS SNPs causing amino acid variations (AAVs), and 1768 gene absence/presence variations (GAPs). All AAVs and GAPs (*n* = 19,404) were assessed for association with meningitis using the LMM, with population structure based on all 33,710 CDS SNPs and patient age group as a covariate (Fig. 2b). Model fitting using the FaST-LMM software showed that the candidate variant *pbp1b*641C was significantly associated with meningitis (*P* = 2.28 × 10^−6^) and was again the most significant hit (Fig. 2c). Model fitting using the GEMMA software generated similar results (Supplementary Table 19). Variants at loci adjacent to the *pbp1b* gene showed no significant association with meningitis and a low level of linkage disequilibrium with the *pbp1b*641 locus (Fig. 2d). In this cohort, 23.6% (42) meningitis patients and 10.6% (247) non-meningitis patients were infected by the *pbp1b*641C genotype pneumococci. Among the 2229 non-*pbp1b*641C isolates, 99.8% (2225) had sequence A at the position *pbp1b*641 while 0.2% (4) had a missing value.

To complement the assembly and COG-based variant call approach and to enhance the power of identifying additional candidate variants, we pooled the exploratory and the confirmatory isolates for a combined screening of genome-wide SNPs, which were called from short-reads mapping against a reference TIGR4 genome (AE005672.3; [https://www.ncbi.nlm.nih.gov/nuccore/AE005672.3]). After quality control, 57,765 genome-wide SNPs were identified from the combined datasets, of which 12,096 were non-synonymous. The association between non-synonymous SNPs and meningitis was assessed using the LMM with population structure calculated based on all 57,765 genome-wide SNPs, and patient age group as a covariate (Supplementary Figure 1). Model fitting showed that the proportion of phenotype variance explained by pneumococcal genetic variation was 0.0571 (95% CI: 0.0263–0.0927). The *pbp1b*A641C SNP (TIGR4 genome T2008526G) remained as the most significant candidate (*P* = 2.99 × 10^−10^) and the association was genome-wide significant after a Bonferroni correction of the *P*-value (corrected *P* = 1.73 × 10^−5^). No additional candidate was identified based on Bonferroni-corrected *P*<0.05 (Supplementary Figure 1). The linkage disequilibrium (r^2^) between the *pbp1b*641 locus and the other 12,095 non-synonymous SNP loci ranged from 0 to 0.44 (Supplementary Figure 1). A maximum-likelihood phylogenetic tree of all isolates labeled with disease status and candidate variant is shown in Supplementary Figure 2.

### Functional implication of the *pbp1b*641C mutation

**Fig. 3:**
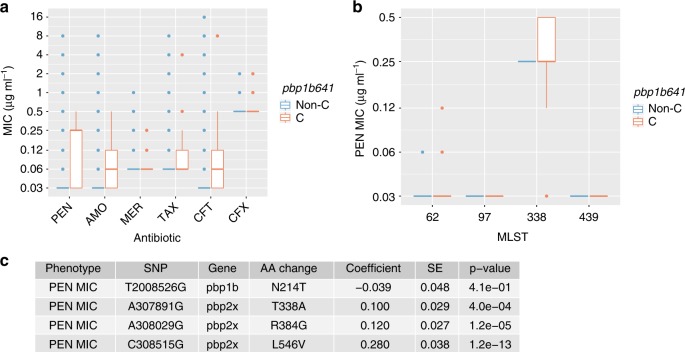
Effects of *pbp1b*A641C on antibiotic resistance in clinical isolates. **a** Comparison of antibiotic MIC distribution between the non-*pbp1b*641C isolates (blue) and *pbp1b*641C isolates (orange) in the confirmatory cohort. PEN penicillin, AMO amoxicillin, MER meropenem; TAX cefotaxime, CFT ceftriaxone, CFX cefuroxime. **b** Comparison of PEN MIC distribution between the non-*pbp1b*641C isolates (orange) and *pbp1b*641C isolates (blue) within the same multi-locus sequence type (MLST). The four MLSTs containing both *pbp1b*641C and non-*pbp1b*641C isolates are shown. In all boxplots, center line is the median and the two box bounds are the first and third quartile. A whisker is 1.5-interquartile range from the closest box bound. Data outside the whiskers are plotted as dots. **c** Association between the *pbp1b*A641C variant and increased PEN MIC (log2 transformed) assessed by a linear mixed-effects model (LMM) controlling for population structure. SNPs are relative to reference TIGR4 genome. SNP T2008526G corresponds to the *pbp1b*A641C mutation and the others three SNPs are known to conferring β-lactam resistance. Coefficient indicates the increase in log_2_(PEN MIC) associated with the alternative allele compared to the reference allele. SE is the standard error of the coefficient. Coefficients, SEs, and *P*-values were derived from fitting the MIC and genotype data to the LMM

The *pbp* gene family is involved in conferring resistance to β-lactam antibiotics in pneumococcus. Major resistance determinants are changes in the PBP1a, PBP2b, and PBP2x proteins that resulted in reduced affinity for the antibiotics^24^. Other changes both within and outside the PBPs presumably compensate for fitness costs^24^. To explore the potential role of the *pbp1b641C* mutation in β-lactam resistance, the confirmatory cohort isolates were tested for minimum inhibitory concentration (MIC) to six β-lactam antibiotics commonly used to treat pneumococcal infections (penicillin, amoxicillin, meropenem, cefotaxime, ceftriaxone, and cefuroxime). A total of 1716 and 13,332 MIC values were obtained for the 286 *pbp641*C clinical isolates and 2222 non-*pbp641*C clinical isolates, respectively (Supplementary Data 2). Sixty MIC values were missing from 10 clinical isolates (three *pbp641*C isolates and seven non-*pbp641*C isolates; Supplementary Data 2). MIC values of the six antibiotics were highly correlated, with pairwise correlate coefficients ranging from 0.84 to 0.95 (Supplementary Figure 3). The *pbp641*C clinical isolates and non-*pbp641*C clinical isolates showed equal median MIC for meropenem (0.06 µg ml^−1^), cefotaxime (0.06 µg ml^−1^), and cefuroxime (0.5 µg ml^−1^) (Fig. 3a), while the median MIC of *pbp641*C clinical isolates was higher than that of the non-*pbp641*C clinical isolates for penicillin (0.25 vs. 0.06 µg ml^−1^), amoxicillin (0.06 vs. 0.03 µg ml^−1^), and ceftriaxone (0.06 vs. 0.03 µg ml^−1^) (Fig. 3a). Only for penicillin, the difference in median MIC was more than one two-fold dilution. Within the same MLST, however, *pbp641*C and non-*pbp641*C isolates showed equal median MIC for penicillin (Fig. 3b), suggesting the *pbp641*C mutation might be associated with β-lactam-resistant lineages but was not a sufficient cause of substantially increased MIC. Analysis of a linear mixed-effects model on penicillin MIC, incorporating fixed effect for *pbp1b641*C and random effects for population structure based on genome-wide SNPs, indicated no independent contribution to increased MIC from *pbp1b*641C (mean increase in log2(MIC) = −0.039, SE = 0.048, *P* = 0.41). In contrast, PBP changes known to be implicated in β-lactam resistance (PBP2X T338A, R384G, and L546V) showed highly significant association with increased penicillin MIC after controlling for population structure (Fig. 3c). Similar results were also observed for the other five β-lactam antibiotics (Supplementary Tables 1–6, Supplementary Figure 4). Thus, the *pbp1b*641C mutation was unlikely to cause a β-lactam-resistant phenotype.

**Fig. 4:**
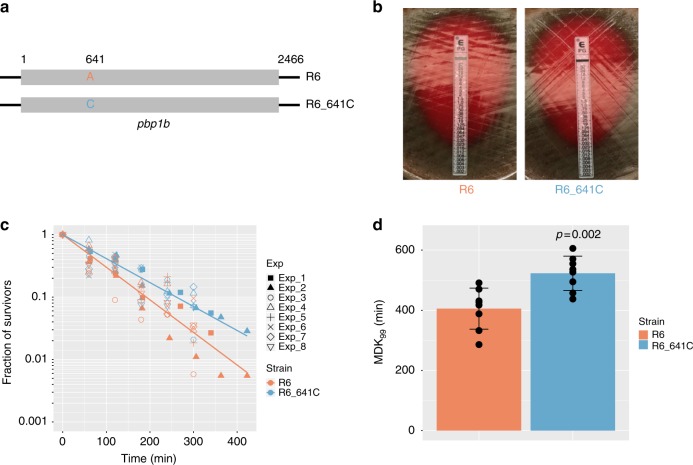
Effects of *pbp1b*A641C on antibiotic resistance and tolerance in isogenic strains. **a** The *pbp1b* locus in the R6 strain was replaced by an allele carrying the *pbp1b*A641C point mutation to construct the R6_641C strain. **b** MIC test carried out using the penicillin E-test strip. **c** Survival of the isogeneic R6 and R6_641C strains in the presence of 3 μg ml^-1^penicillin (> 100-fold MIC). Data from eight independent experiments (Exp_1 to Exp_8) are shown. **d** Minimum duration for killing 99% of bacterial cells in the population (MDK_99_) estimated from the survival curves. Data are shown as mean ± SD from the eight experiments in **c**. Dot plots are overlaid in the bar charts to show MDK_99_ estimation from individual experiments. The *P*-value is based on a two-sample *t* test (two tailed)

To further explore biological effects of the *pbp1b*641C genotype, we constructed a pair of isogenic laboratory strains, R6 and R6_641C (Fig. 4a). The *pbp1b*641A allele in the parental R6 strain was replaced by a *pbp1b*641C allele to create the R6_641C strain that reproduced the candidate variation. Whole-genome sequencing confirmed the expected *pbp1b*641 allele sequence in each strain and did not find spurious mutations. Antibiotic MICs of R6 and R6_641C were equivalent for penicillin (Fig. 4b, 0.023 µg ml^−1^) and 17 other antibiotics used for IPD management (Supplementary Table 25). No antibiotic resistance resulted from the *pbp1b*641 A to C change (Supplementary Table 25). We next compared the level of antibiotic tolerance between R6 and R6_641C by estimating the typical duration of penicillin treatment that was needed for killing 99% of bacterial cells in the population (MDK_99_). In the presence of 3 µg ml^−1^ penicillin (> 100-fold MIC), the R6_641C strain showed a substantially slower rate of death compared with the parental R6 strain (Fig. 4c). The estimate MDK_99_ of the R6_641C strain was 522 ± 57 min (mean ± SD; Fig. 4d), which was significantly longer than that of the parental R6 strain (405 ± 68 min; *P* = 0.002, two-sample *t* test, two tailed, t = 3.73, df = 13.57; Fig. 4d).

Antibiotic tolerance is thought to enhance bacterial cell survival through a transient exposure to lethal concentrations of antibiotic, a scenario commonly encountered by the pneumococci in both carriage and disease. We therefore assessed whether the *pbp1b*641 A to C substitution in clinical isolates was selectively neutral or was associated with selection advantage by analyzing the inferred evolutionary history of the *pbp1b* gene. We first identified that bases 816–1028 of *pbp1b* were likely affected by horizontal transfer (Supplementary Figure 5A) and masked this region before inferring a phylogeny of the *pbp1b* gene using the remaining putative point mutations. The *pbp1b*641C genotype was observed in six distinct clades of the inferred *pbp1b* gene phylogeny, each corresponding to different MLSTs (Supplementary Figure 5B). Each of the six clades contained an independent *pbp1b*641 A to C substitution as they each evolved from a common ancestor of the A genotype. Only 10 of the 2466 bases in *pbp1b* showed six or more substitutions events in the inferred *pbp1b* gene phylogeny (Supplementary Figure 5C), suggesting the *pbp1b*641 A to C substitution belonged to a rare group of changes that had been repeatedly acquired by different lineages. Further, the non-synonymous substitution rate (dN) for codon214 was significantly higher than the synonymous substitution rate (dS) of the same codon site (Supplementary Figure 5D). Analyzing the inferred phylogeny using a different evolutionary model (mixed-effects model of evolution, MEME) showed similar evidence that dN was greater than dS for codon214 (Supplementary Figure 6), suggesting the codon site was under diversifying positive selection.

### Effect size estimation for the *pbp1b*641C mutation

We estimated the effect size of *pbp1b*641C on meningitis using a mixed-effects logistic regression model to explicitly account for well-characterized pneumococcal phenotypes potentially associated with virulence (serotype and antibiotic resistance) as well as patient age.

Fitting the model to the confirmatory dataset indicated that patients infected by *pbp1b*641C pneumococci had an adjusted odds ratio (aOR) of 2.83 (95% CI: 1.65–4.84) for meningitis compared with patients infected by non-*pbp1b*641C pneumococci. Compared with patients aged 18–64, patients aged < 2 years showed higher risk of meningitis (aOR = 1.97, 95% CI: 1.10–3.38) while patients aged > 64 years exhibited lower risk (aOR = 0.46, 95% CI: 0.32–0.66). We concluded that *pbp1b*641C appeared to be an independent predictor of meningitis among IPD patients and its effect size was comparable with that of patient age.

**Fig. 5:**
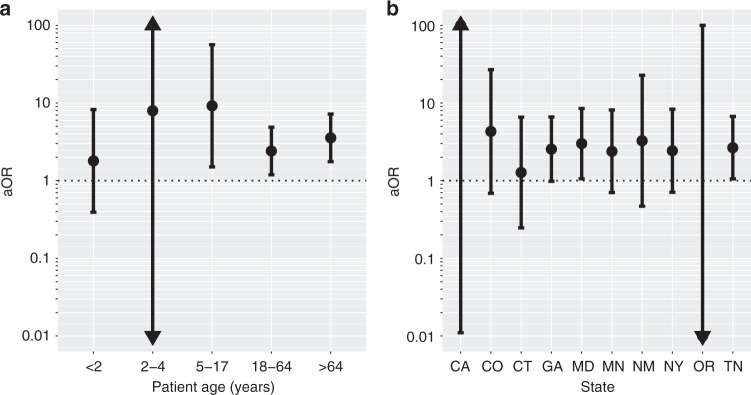
Effect size of the *pbp1b*641C genotype on meningitis syndrome in the confirmatory cohort. **a** Effect size stratified by patient age group. **b** Effect size stratified by surveillance state. Solid dot indicates the adjusted odds ratio (aOR) of *pbp1b*641C estimated from a mixed-effects logistic regression model explicitly accounting for pneumococcal serotype (random effects), and susceptibility to six β-lactam antibiotics (random effects). Error bars are 95% confidence intervals. An arrow indicates the boundary of the estimated value is beyond the *y*-axis limit

Next, we examined whether the association between *pbp1b*641C and meningitis was driven by a particular epidemiological subgroup (Fig. 5). The isolates were stratified by patient age range and surveillance site (state). For each stratum, the effect size of *pbp1b*641C was estimated using the above mixed-effects logistic regression model. There appeared to be a trend of stronger association in patients aged 5–17 and > 64 years (Fig. 5a), and in states other than Oregon and Connecticut (Fig. 5b). However, no evidence of significant variation in *pbp1b*641C effect size among subgroups was found (all F-tests *P* > 0.05), suggesting lack of SNP interactions with age group or surveillance site.

In addition, we analyzed the above mixed-effects logistic regression model incorporating fixed effects of individual antibiotic, either as a binary variable (susceptible vs. non-susceptible) or as a continuous variable (MIC values). In all analyses, *pbp1b*641C remained significantly associated with meningitis after adjusting for patient age, serotype, and individual antibiotic resistance. The aORs of *pbp1b*641C ranged from 2.46 to 3.09 (Tables S8–S19). Individual antibiotic non-susceptibility was significantly associated with meningitis except for Penicillin (Tables S8–S13), and their aORs ranged from 1.50 (penicillin) to 2.59 (amoxicillin). Higher MIC were significantly associated with meningitis for all six antibiotics (Supplementary Tables 12–17), and the aORs per one unit increase in log_2_(MIC) ranged from 1.13 (penicillin) to 1.55 (cefuroxime).

### Distribution of the *pbp1b*641C allele

The *pbp1b*641C allele was found in 9.5% (434) of IPD isolates examined in this study. This allele was widely distributed among 18 serotypes and 54 MLSTs. Serotypes with high proportion of *pbp1b*641C allele were 5 (6/6), 10 A (69/82), 23 A (138/164), 23B (103/127), 11 A (74/118), 10 F (2/2), and 23 F (22/58). The ABCs 2015 IPD isolates were used to estimate the current distribution of *pbp1b*641C by serotypes belonging to different vaccines, patient age, and geographic location. In this cohort, 289 isolates (11.5%) carried the *pbp1b*641C allele, with vast majority of the allele (288/289) found in the 1937 non-PCV13 serotype isolates. More than a third of the *pbp1b*641C substitution (104/289) occurred within the 1602 isolates, whose serotypes were included in the 23-Valent Pneumococcal Polysaccharide Vaccine (PPSV23). Among patients aged < 2, 2–4, 5–17, 18–64, and > 64 years, the *pbp1b*641C frequency was 0.16, 0.22, 0.12, 0.11, and 0.11, respectively, and the difference was not significant (Fisher’s exact test, *P* = 0.085). The frequencies of *pbp1b*641C allele observed in the ten states were 0.06 (NM), 0.07 (CA), 0.09 (CO), 0.09 (MN), 0.13 (CT), 0.13 (TN), 0.14 (MD), 0.14 (NY), 0.14 (OR), and 0.15 (GA). The variation by state was modest yet significant (Fisher’s exact test, *P* = 0.0035).

## Discussion

In this study, we identified a *pbp1b*A641C variant that was associated with meningitis after controlling for patient age and population structure in an exploratory sample. The higher frequency of *pbp1b*641C allele among isolates from meningitis patients was subsequently validated in a sufficiently-powered, independent confirmatory cohort. This finding raised two potential hypotheses on how the presence of *pbp1b*641C allele may lead to increase in meningitis risk: (1) that the *pbp1b*641C genotype by itself increases the potential of pneumococcus to invade human meninges during infection, and (2) that the *pbp1b*641C genotype is associated with other factors that could increase the relative frequency of meningitis among ABCs IPD cases. Despite being a well-known risk factor for bacterial meningitis, patient age was unlikely to explain the effect of *pbp1b*641C because the frequency of *pbp1b*641C was similar among all age groups. Although pneumococcal serotype and β-lactam resistance, two major virulence determinants, showed association with the *pbp1b*641C allele, explicitly controlling for these covariates did not abolish the significant association between *pbp1b*641C and meningitis. These results collectively suggested that presence of the *pbp1b*641C allele could serve as an independent bacterial predictor of meningitis among IPD patients.

The PBP1b protein belongs to the class A PBPs that contain two function domains: transglycosylase and transpeptidase. Both enzymatic activities contribute to the synthesis of bacterial cell wall peptidoglycan, with the former responsible for glycan strand elongation and the latter for peptide chain cross-linking. The β-lactam antibiotics kill bacteria through binding to and inhibiting the activity of the transpeptidase. Nearly all pneumococcal isolates carried the *pbp1b* gene although it is not required for in vitro growth^25^. In exploring the functional consequence of the *pbp1b*641C mutation, we found no evidence that it caused β-lactam resistance, consistent with previous reports^26^. Nonetheless, the *pbp1b*641 A to C mutation resulted in longer duration needed for killing by penicillin in isogeneic laboratory strains, and the codon site containing the *pbp1b*641 A to C substitution showed evidence of being under positive selection in clinical strains. We therefore hypothesized that the mechanism underlying the association was possibly due to a survival advantage from the increased antibiotic tolerance that was separable from β-lactam resistance. For example, *pbp1b*641C may alter the activity of transglycosylase domain to allow bacteria surviving transient exposure to antibiotics despite being susceptible, thus providing more opportunities for their translocation from the blood stream to the brain. It should be noted that pneumococci are fundamentally and primarily commensal organisms colonizing human nasopharynx. Similar to that IPD is often an accidental outcome of colonization, the increased virulence associated with *pbp1b*641C could be an unintended consequence of certain fitness benefits for commensal lifestyle, and might not be the cause of the selection advantage.

The *pbp1b*641C seemed to be compatible with many pneumococcal genetic backgrounds, indicated by its broad distribution among serotypes and lack of strong linkage disequilibrium with other genome-wide SNP loci. The *pbp1b*641C allele frequency is likely to further increase due to reduced competition from the diminishing PCV13 serotypes, which tended to not having the mutation. By comparison, PPSV23 serotypes covered a much higher proportion (36%) of the *pbp1b*641C isolates. If patient infected by pneumococci of this genotype were proved to be at higher risk of developing meningitis, populations with high *pbp1b*641C prevalence could potentially garner additional benefits from vaccine-based prevention efforts.

Limitations of this study include that the sample size, although large, is underpowered to evaluate the large number of rare mutations and gene–gene interactions that may facilitate the development of meningitis, given the conservative GWAS significance threshold. Ascertainment biases and potential confounding could be present in both sample sets, which may have limited our power to evaluate rarer variants. The cohort design used in the confirmatory study reduced potential selection biases associated with a case-control design but limited the number of meningitis cases due to its low incidence rate. Because of years of PCV13 use in the United States, we may not have had sufficient PCV13 serotype isolates to evaluate *pbp1b*641C frequency among them in year 2015. Due to incomplete annotation of draft genome assemblies, certain paralogs could have been misclassified as orthologues and genes that tend to break contigs could have been misclassified as absent in some isolates, although the scope of the problems appeared to be limited. In addition, sequence variation in the form of insertion/deletion (idnel) was not assessed and could be explored by a k-mer-based approach.

In summary, by screening both the core and accessory genes in pneumococcal isolates from IPD patients, we identified a replicable bacterial genetic variant (*pbp1b*641C) associated with clinical manifestation of meningitis. We also observed that adjustment for patient age, geographic location, pneumococcal serotype, or β-lactam resistance minimally affected the strength of this association, indicating a robust correlation. Other unique findings of this study include evidence of selection advantage associated with the *pbp1b*641C allele and description of its prevalence in serotypes covered by current pneumococcal vaccines. Host, pathogen, and environmental factors probably all contribute to the complex manifestations of infectious disease. Much remains to be learned about the microbiological determinants of disease risk, transmission, and prognosis in humans. Advances in this filed hold great potential for improving public health.

## Methods

### Clinical isolates and characterization

Invasive pneumococcal isolates were obtained through the ABCs. ABCs is an active, population- and laboratory-based surveillance system that is part of the Centers for Disease Control and Prevention’s (CDC) Emerging Infections Program. Cases of invasive pneumococcal diseases (IPD) were defined as the isolation of pneumococci from normally sterile sites in residents of the surveillance areas in ten different states^3,27,28^. The surveillance areas represent 33,761,932 residents as of 2014^23^.

ABCs case reporting and isolate collection were regarded as surveillance activities and were either exempt from institutional review or approved by institutional review boards^29^. Informed consent is not required by institutional review boards at CDC or individual surveillance site^29^.

The exploratory sample consisted of 2054 ABCs isolates whose time of isolation ranged from 1995 to 2013. These isolates broadly represented ABCs cases reported during these years (Supplementary Table 20). Younger patients were oversampled into the exploratory sample, with total of 68.3% isolates from patients aged < 5 years. This proportion was much higher than the proportion of all IPD cases from patients aged < 5 years (9.1–25.1%) in these surveillance years. The confirmatory cohort consisted of 2518 isolates obtained in year 2015 surveillance that had whole-genome sequencing data and disease manifestation information (meningitis vs. non-meningitis) available as of June 1, 2016. These 2518 isolates represented 85.6% of all IPD cases in the surveillance population in year 2015. For all isolates, information on meningitis diagnosis, patient age, and sample year were obtained from the patient’s chart as per routine ABCs procedures. Meningitis case was defined as isolation of pneumococci from cerebrospinal fluid (CSF) or clinical diagnosis of meningitis and blood or other sterile site isolate. Strain features were obtained from laboratory characterizations^21,22,30^. Serotype was determined by Quellung tests and WGS-based predictions^21^. For confirmatory cohort isolates, minimum inhibitory concentrations (MICs) of six β-lactam antibiotics (penicillin, amoxicillin, meropenem, ceftriaxone, cefotaxime, and cefuroxime) were determined by the broth microdilution method. MIC interpretive standards are shown in Supplementary Table 21 and are consistent with Clinical and Laboratory Standards Institute (CLSI) document M100-23.

### Whole-genome sequencing and CDS variant calling

All isolates were sequenced on the Illumina platform^21,22^ with a median coverage of 212 × and 104 × for the exploratory sample and confirmatory cohort, respectively. Following removal of adaptor sequences and low-quality bases (q score < 20), the Illumina short-reads of each isolate were assembled de novo using the Velvet software with a k-mer size determined by VelvetOptimiser^31^. Putative CDSs and the encoded protein sequences were extracted from the assembled contigs using Prodigal v2.60^32^ with the -c option to exclude CDSs running off contig edges. To identify cluster of orthologue groups (COGs), all predicted protein sequences from the exploratory sample (*n* = 4,485,989) were combined with 41,339 reference protein sequences from 20 annotated, complete pneumococcal genomes (Supplementary Table 22). The reference protein sequences were included to aid COG annotation. A k-mer based sequence clustering algorithm, MMseqs^33^, was used to cluster the combined sequences into 4724 homologous and functionally similar clusters, each containing 20 or more sequences and showing a minimum of 70% sequence within-cluster identity. The longest sequence in a cluster was identified as the representative sequence for the cluster. If a cluster contained two or more protein sequences from the same isolate, only the isolate sequence showing the highest alignment matches with the representative sequence was retained. After removing 52694 (1.2%) redundant sequences, we defined each cluster as a COG, to which each isolate contributed either 0 or 1 CDS, indicating gene absence or presence, respectively. Protein sequences in each COG were globally aligned using Clustalo and back-translated into DNA sequence alignment using PAL2NAL^34,35^. Synonymous and non-synonymous SNPs in each CDS were called from the alignments using a custom script. CDS SNPs and gene absence/presence variations (GAPs) were coded as haploid human mitochondrial genotype using the PLINK software (version 1.07)^36^. Only the two most frequently observed alleles at each variation site were retained. SNPs with minor allele frequency (MAF) < 0.05 or information missing from more than 5% of isolates (missingness > 0.05) were filtered out because of limited test power. Similarly, GAPs representing gene presence in < 5% or > 95% of isolates were filtered out. The same COG identification and variant calling procedure was applied to the analysis of confirmatory cohort isolates.

To rapidly identify the *pbp1b641C* candidate variant, we created a database of variant alleles (Supplementary Table 23) and used the SRST2 software^37^ to perform sequence typing based on short-reads mapping. For the 2038 exploratory sample isolates in which the *pbp1b*641 sequence was either A or C as determined by the de novo assembly and clustering method, short-read sequence typing results were 100% concordant. We therefore used the short-read sequence typing result of *pbp1b641C* for all isolates.

### Genome-wide association analysis of individual dataset

LMM implemented in the FasT-LMM software^38^ was used in all association studies unless otherwise specified. Four input data files were prepared from each dataset as described below. First, the phenotype file was generated using the PLINK software^36^, in which phenotype was coded as a binary variable (meningitis vs. non-meningitis). Second, the test variant file consisted of gene absence/presence variations GAPs and non-synonymous CDS SNPs indicating amino acid variations (AAVs). GAPs and AAVs were selected because they were more likely to result in altered cellular functions that facilitate infecting meninges compared with synonymous CDS SNPs. Third, the SNP data file used to determine the genetic similarities between isolates consisted of all CDS SNPs. Fourth, the covariate file was patient age group (< 2, 2–4, 5–17, 17–64, and > 64 years).

The LLM model was also fitted by an implementation in the GEMMA software^39^ using the same four input files. In both software, the population structure was calculated by a pairwise matrix indicating genome-wide SNPs between isolates provided by the input data file.

### Power analysis for confirmatory study

Population structure- and age-adjusted coefficient estimate for *pbp1b641C* (*β*_*e*_ = 0.02674) and its standard error (*se* *=* 0.005927) from the FasT-LMM fitting of the exploratory sample were used for power estimation. The *pbp1b641C* coefficient estimate from a replication study (*β*_*r*_) of the same sample size (*n* = 2054) was assumed to follow a normal distribution with a mean of *β*_*e*_ and a standard deviation of *se*. The null hypothesis was that true *pbp1b641C* coefficient was 0, thus *βr* ~ N(0, *se*^2^). The power of the replication study to reject the null hypothesis at significance level of 0.05, 0.01, and 10^-4^ was approximated by the probability of observing a *βr* that is greater than 1.96*se*, 2.56*se*, and 4.89*se*, respectively, given true *βr* ~ N(*β*_*e*_, *se*^2^).

### Combined SNP analysis

Reads from isolates of both datasets were mapped against a single reference genome, TIGR4 (AE005672.3; [https://www.ncbi.nlm.nih.gov/nuccore/AE005672.3]), using the bowtie2.1.0 software^40^. SNPs were called from mapped sequences using the samtools0.1.19 software with the following filters: mapping quality score > 30, coverage depth > 10, and alternative allele frequency = 1. The SnpEff software^41^ was used to annotate synonymous and non-synonymous SNPs. SNPs with MAF < 0.05 or missingness > 0.05 were filtered out. The number of SNPs identified in the combined analysis were higher compared with the individual dataset because: (1) reads mapping is presumably more sensitive for variant calling compared with the de novo assembly approach and (2) the combination of exploratory and confirmation datasets resulted in a more diverse population which harbored more SNPs than each individual dataset. SNPs were coded as a haploid human mitochondrial genotype using PLINK and analyzed using an LMM similar to what was described above. Genetic similarities between isolates were calculated using all genome-wide SNPs and were incorporated in the LMM as random effects. Patient age group (< 2, 2–4, 5–17, 17–64, and > 64 years) was incorporated in the model for fixed effects. The association between meningitis and all non-synonymous SNPs was tested. A Bonferroni-corrected *P* < 0.05 was used as the threshold for identifying candidate variants. Heritability and its confidence intervals were estimated using the ALBI (Accurate LMM-based heritability Bootstrap confidence Intervals) software^42^. Linkage disequilibrium (r^2^) between the *pbp1b*641 locus (TIGR4 genome position2008526) and all non-synonymous SNP loci was calculated by PLINK. A maximum-likelihood phylogenetic tree of all isolates was inferred using the FastTree2.1.8 software^43^ based on concatenation of genome-wide SNPs and the generalized time-reversible nucleotide substitution model.

### Effect size estimation

The effect size of the candidate variant on meningitis was estimated by fitting a mixed-effects logistic regression model that explicitly controlled for pneumococcal serotype, antibiotics susceptibility, and patient age group. In this analysis, population structure was approximated by serotype because: (1) we intended to explicitly adjust for the most important pneumococcal virulence factor; (2) the study sample covered majority (54) of the more than 90 diverse pneumococcal serotypes; and (3) serotypes have been shown to correlate well with clonal lineages defined by genome-wide SNPs in multiple large studies^42,44,45^. The model incorporated a binary outcome (meningitis vs. non-meningitis) and fixed-effects for the candidate variant. Non-susceptibility to each antibiotic was combined into a barcode to represent the covariate antibiotics susceptibility. For example, an isolate susceptible (S) to all six β-lactam antibiotics would be coded as SSSSSS, while an isolate non-susceptible (N) to penicillin but susceptible to all the other five β-lactam antibiotics would be coded as NSSSSS. Serotype and antibiotics susceptibility were incorporated in the model for random effects because: (1) the observed levels of covariate (serotypes or combinations of susceptibility status) were a sample of all possible levels and (2) sample size for individual levels was small and some levels contained zero count of either meningitis case or the candidate variant. Covariate age group ( < 2, 2–4, 5–17, 17–64, and > 64 years) was incorporated in the model for fixed effects. The confirmatory cohort data were used to for effect size estimation because it represented a well-defined population. Model fitting was performed using the lme4 package in R. Adjusted odds ratio (aOR) of candidate variant and its 95% confidence interval (CI) were computed as estimations of increased meningitis risk level associated with the candidate variant (effect size). In subgroup analysis, the confirmatory cohort was stratified by age group or geographic location. Subgroup-specific aOR and 95% CI were computed using the lme4 package in R. In separate mixed-effects logistic regression models, susceptibility or MIC of an individual antibiotic was also incorporated as fixed effects to evaluate their effect size.

### Phylogenetic analysis of *pbp1b* sequences

Non-redundant CDSs in the COG corresponding to the *pbp1b* gene (*n* = 326) were globally aligned based on the encoded amino acid sequences using Clustalo and PAL2NAL^34,35^. The codon-alignment was scanned by the Gubbins algorithm^46^ to identify regions containing elevated densities of base changes indicating horizontal sequence transfer. A maximum-likelihood phylogeny was concurrently constructed by Gubbins based on the putative point mutations outside regions of high sequence diversity. Number of independent base substitution at each site was inferred from the phylogeny. To assess whether the non-synonymous substitution rate (dN) at a specific codon site is higher than the synonymous substitution rate (dS) at the same site, the codon-alignment, after removing codon sites within regions affected by horizontal sequence transfer, and the phylogeny was analyzed by a Bayesian approximation method implemented in the FUBAR software^47^. The posterior codon-specific distributions for the selection parameters were inferred from five independent Markov chain Monte Carlo (MCMC) runs. Each MCMC chain had a length of 2 × 10^6^ with the first half discarded as burn-in. A codon site with a posterior probability of (dN > dS) greater than 0.95 was defined as showing evidence of being under diversifying/positive selection. The partitioned codon-alignment was also analyzed by a mixed-effects model of evolution (MEME)^48^ to identify instances of both episodic and pervasive positive selection at the level of an individual codon site. Both FUBAR and MEME analyses were performed using the HyPhy package^49^.

### Antibiotic susceptibility in clinical isolates

The potential effects of *pbp1b*641C mutation on non-susceptibility to six β-lactam antibiotics were assessed using the confirmatory cohort data and a LLM model similar to what was used for genome-wide association analysis of meningitis. Antibiotic MIC values obtained from the broth microdilution tests were used as continuous outcome data. In addition to the *pbp1b*641C mutation, three variants known to reduce antibiotic affinities to the targets (PBP2X T338A, R384G, and L546V)^24^ were also tested for association with increased MIC as positive controls. The allelic information of these three variants were obtained from short-reads mapping against the reference TIGR4 genome as described above. Genetic similarities between isolates based on genome-wide SNPs were incorporated in the LMM as random effects to control for bacterial population structure. The models were fitted by the FasT-LMM software^38^. A variant with *P*-value < 0.05 was considered to be independently associated with increased MIC of the antibiotic and thus a likely causal variant for resistance.

### Characterization of *pbp1b*641C isogenic laboratory strains

Laboratory strains of *S. pneumoniae* and PCR primers used in this study are listed in Supplementary Table 24. All strains were maintained in Todd–Hewitt broth supplemented with 0.5% yeast extract (THY) or trypticase soy agar plate with 5% sheep blood (TASII; Becton, Dickinson and Company, MD). Genetic transformations of *S. pneumoniae* strains were performed according to the method described by Pozzi et al.^50^. Competence Stimulating Peptide-1 (CSP-1, final concentration 500 ng ml^−1^) was used in all transformations. Selective reagent concentrations in selective media were 500 µg ml^−1^ for kanamycin, 200 µg ml^−1^ for streptomycin, and 10% w/v for sucrose. The concentration of donor DNA used in transformation were 875 ng ml^−1^ for PCR products. Strains were stored in THY supplemented with 10% glycerol at −80 °C.

To make the R6 *pbp1b*641C isogenic strain, a streptomycin-resistant R6 strain^51^ was transformed to replace its original *pbp1b*641A allele (Spr1909 [https://www.ncbi.nlm.nih.gov/gene/?term = Spr1909], 2466nt) with a *pbp1b*641C allele through a Sweet-Janus-mediated, two-step exchange^52^. In the first step, primers YL8001 and YL8002 were used to amplify an 1101-bp fragment upstream of the *pbp1b* locus corresponding to 1885293 to 1886393 of the R6 reference genome NC003098.1[https://www.ncbi.nlm.nih.gov/nuccore/NC_003098.1] (NC003098.1:1885293.1886393) using genomic DNA of R6 as a template. Primers YL8005 and YL8006 were used to amplify an 1101-bp fragment downstream of the *pbp1b* locus (NC003098.1: 1888443.1889543) using genomic DNA of R6 as a template. Primers YL8003 and YL8004 were used to amplify a 2807-bp Sweet Janus cassette (1 to 2807 of the cassette sequence KJ845726.1 [https://www.ncbi.nlm.nih.gov/nuccore/KJ845726.1/]) using genomic DNA of SPNYL001^52^ as a template. These three fragments were ligated together using the NEBuilder HiFi DNA Assembly Master Mix (New England Biolabs, MA), and the assembly product was PCR amplified using primers YL8007/8008. The resulting PCR product was used to transform the R6 strain to construct the R6_SJ strain (selected by kanamycin), in which the *pbp1b* locus was replaced by the Sweet-Janus cassette. In the second step, primers YL8001 and YL8010 were used to amplify a 2500-bp fragment from R6 genomic DNA (NC003098.1:1887842.1885293) containing nucleotides 623–2466 of the *pbp1b* coding sequence (on the negative strand) and adjacent upstream genome sequence of the *pbp1b* locus, with a *pbp1b*A641C mutation introduced by primer YL8010. Primers YL8006 and YL8009 were used to amplify a 1666-bp fragment from R6 genomic DNA (NC003098.1:1889543.1887878) containing nucleotides 1–659 of the *pbp1b* coding sequence and adjacent downstream genome sequence of the *pbp1b* locus, with a *pbp1b*A641C mutation introduced by primer YL8009. These two PCR fragments were ligated together using the NEBuilder HiFi DNA Assembly Master Mix, and the assembly product was PCR amplified using primers YL8007/8008. The resulting PCR product was used to transform the R6_SJ strain to construct the R6_641C strain (selected by streptomycin and sucrose), in which the SweetJanus cassette was replaced by the mutant *pbp1b* gene sequence.

Whole-genome sequencing and short-read sequence typing of laboratory strains were performed using the same methods as described for the clinical isolates. Antibiotic MICs were determined by using E-test plastic strips (bioMérieux, NC) and broth microdilution plates (Thermo Fisher Scientific Trek, MA) according to manufacturers’ instructions. Antibiotic tolerance was evaluated based on time-kill curves^53,54^. Briefly, strains were streaked onto TSAII plates and incubated at 37 °C in a 5% CO_2_-enriched atmosphere for 16 to 24 h. Colonies from the plate were harvested into THY supplemented with 3 mg/l penicillin (> 100-fold MIC) to a turbidity equivalent to 0.5 McFarland standard (~1 × 10^7^ CFU ml^−1^) and cultured at 37 °C in a 5% CO_2_. At indicated time points, aliquots of the culture were diluted to the appropriate concentrations in THY and plated out onto TSAII plates. Colony forming units (CFUs) were counted from the plates after overnight incubation and were converted to CFU density in the culture at each time point. The fraction of surviving bacterial population at a time t was calculated by dividing the CFU density at time t by the CFU density at time 0. To estimate the minimum duration for killing 99% of bacterial cells in the population (MDK_99_), the time-surviving fraction data were fitted to a linear regression model, and the MDK_99_ was extracted from the model using the inverse.predict function in the chemCal package of the R software^55^.

### Statistics

The LMM was used for GWAS as described above. Association between two categorical variables was evaluated by the Fisher’s exact test. Confidence intervals for proportions were constructed using the exact binomial method. Adjusted odds ratios (aORs) and confidence intervals were estimated using mixed -effects logistic regression models. All statistical analyses were performed in FasT-LMM v2.07^38^, GEMMA v0.94.1^39^, PLINK v1.07^36^, R v3.2.2^55^, and HyPhy v2.2^49^. Graphics were created in R 3.2.2^55^.

## Supplementary information

Supplementary Information

Description of Additional Supplementary Files

Supplementary Data 1

Supplementary Data 2

## Data Availability

All WGS raw reads data are deposited in European Nucleotide Archive (ENA) and National Center for Biotechnology Information Sequence Read Archive (NCBI). Accession numbers are listed in Supplementary Data files 1 and 2. All other data reported in the paper are included in the paper and Supplementary Materials.

## References

[1] Henriques-Normark, B. & Tuomanen, E. I. The pneumococcus: epidemiology, microbiology, and pathogenesis. *Cold Spring Harb. Perspect. Med.* **3**, dx.doi.org/10.1101/cshperspect.a010215 (2013). 10.1101/cshperspect.a010215 PMC3685878 23818515

[2] Moore MR (2016). Effectiveness of 13-valent pneumococcal conjugate vaccine for prevention of invasive pneumococcal disease in children in the USA: a matched case-control study. Lancet Respir. Med.

[3] Moore MR (2015). Effect of use of 13-valent pneumococcal conjugate vaccine in children on invasive pneumococcal disease in children and adults in the USA: analysis of multisite, population-based surveillance. Lancet Infect. Dis..

[4] O’Brien KL (2009). Burden of disease caused by Streptococcus pneumoniae in children younger than 5 years: global estimates. Lancet.

[5] Drijkoningen JJ, Rohde GG (2014). Pneumococcal infection in adults: burden of disease. Clin. Microbiol. Infect..

[6] Thigpen, M. C. et al. Bacterial meningitis in the United States, 1998–2007. *N. Engl. J. Med*. **364**, 2016–2025 (2011). 10.1056/NEJMoa1005384 21612470

[7] Navarro-Torne A (2015). Risk factors for death from invasive pneumococcal disease, Europe, 2010. Emerg. Infect. Dis..

[8] Tunkel AR (2004). Practice guidelines for the management of bacterial meningitis. Clin. Infect. Dis..

[9] Orihuela CJ (2009). Laminin receptor initiates bacterial contact with the blood brain barrier in experimental meningitis models. J. Clin. Invest..

[10] Ring A, Weiser JN, Tuomanen EI (1998). Pneumococcal trafficking across the blood-brain barrier. Mol. Anal. a Nov. bidirectional Pathw. J. Clin. Invest.

[11] Orihuela CJ, Gao G, Francis KP, Yu J, Tuomanen EI (2004). Tissue-specific contributions of pneumococcal virulence factors to pathogenesis. J. Infect. Dis..

[12] Tuomanen E (1996). Entry of pathogens into the central nervous system. FEMS Microbiol. Rev..

[13] Uchiyama S (2009). The surface-anchored NanA protein promotes pneumococcal brain endothelial cell invasion. J. Exp. Med..

[14] Pracht D (2005). PavA of Streptococcus pneumoniae modulates adherence, invasion, and meningeal inflammation. Infect. Immun..

[15] Ribes S (2016). Thioredoxins and methionine sulfoxide reductases in the pathophysiology of Pneumococcal. J. Infect. Dis..

[16] Wellmer A (2002). Decreased virulence of a pneumolysin-deficient strain of Streptococcus pneumoniae in murine meningitis. Infect. Immun..

[17] Kadioglu A, Weiser JN, Paton JC, Andrew PW (2008). The role of Streptococcus pneumoniae virulence factors in host respiratory colonization and disease. Nat. Rev. Microbiol..

[18] Kulohoma BW (2015). Comparative genomic analysis of meningitis- and bacteremia-causing Pneumococci identifies a common core genome. Infect. Immun..

[19] Chewapreecha C (2014). Comprehensive identification of single nucleotide polymorphisms associated with beta-lactam resistance within pneumococcal mosaic genes. PLoS. Genet..

[20] Earle SG (2016). Identifying lineage effects when controlling for population structure improves power in bacterial association studies. Nat. Microbiol.

[21] Metcalf BJ (2016). Using whole genome sequencing to identify resistance determinants and predict antimicrobial resistance phenotypes for year 2015 invasive pneumococcal disease isolates recovered in the United States. Clin. Microbiol. Infect..

[22] Metcalf BJ (2016). Strain features and distributions in pneumococci from children with invasive disease before and after 13-valent conjugate vaccine implementation in the USA. Clin. Microbiol. Infect..

[23] Active Bacterial Core surveillance team. Active Bacterial Core surveillance (ABCs). https://www.cdc.gov/abcs/ (2017).

[24] Hakenbeck R, Bruckner R, Denapaite D, Maurer P (2012). Molecular mechanisms of beta-lactam resistance in Streptococcus pneumoniae. Future Microbiol..

[25] Hoskins J (1999). Gene disruption studies of penicillin-binding proteins 1a, 1b, and 2a in Streptococcus pneumoniae. J. Bacteriol..

[26] Du Plessis M, Smith AM, Klugman KP (2000). Analysis of penicillin-binding protein lb and 2a genes from Streptococcus pneumoniae. Microb. Drug Resist..

[27] Pilishvili T (2010). Sustained reductions in invasive pneumococcal disease in the era of conjugate vaccine. J. Infect. Dis..

[28] Whitney CG (2003). Decline in invasive pneumococcal disease after the introduction of protein-polysaccharide conjugate vaccine. N. Engl. J. Med..

[29] Hampton LM, Zell ER, Schrag S, Cohen AL (2012). Sentinel versus population-based surveillance of pneumococcal conjugate vaccine effectiveness. Bull. World Health Organ.

[30] Li, Y. et al. Penicillin-binding protein transpeptidase signatures for tracking and predicting beta-lactam resistance levels in Streptococcus pneumoniae. *MBio.* **7**, dx.doi.org/10.1128/mBio.00756-16​ (2016). 10.1128/mBio.00756-16 PMC4916381 27302760

[31] Zerbino, D. R. Using the Velvet de novo assembler for short-read sequencing technologies. *Curr Protoc Bioinformatics* **31**, 11–5 (2010). 10.1002/0471250953.bi1105s31 PMC2952100 20836074

[32] Hyatt D (2010). Prodigal: prokaryotic gene recognition and translation initiation site identification. BMC Bioinforma..

[33] Hauser M, Steinegger M, Soding J (2016). MMseqs software suite for fast and deep clustering and searching of large protein sequence sets. Bioinformatics.

[34] Larkin MA (2007). Clustal W and Clustal X version 2.0. Bioinformatics.

[35] Suyama M, Torrents D, Bork P (2006). PAL2NAL: robust conversion of protein sequence alignments into the corresponding codon alignments. Nucleic Acids Res..

[36] Purcell S (2007). PLINK: a tool set for whole-genome association and population-based linkage analyses. Am. J. Hum. Genet..

[37] Inouye M (2014). SRST2: rapid genomic surveillance for public health and hospital microbiology labs. Genome Med..

[38] Lippert C (2011). FaST linear mixed models for genome-wide association studies. Nat. Methods.

[39] Zhou X, Stephens M (2012). Genome-wide efficient mixed-model analysis for association studies. Nat. Genet..

[40] Langmead B, Trapnell C, Pop M, Salzberg SL (2009). Ultrafast and memory-efficient alignment of short DNA sequences to the human genome. Genome Biol..

[41] Cingolani P (2012). A program for annotating and predicting the effects of single nucleotide polymorphisms, SnpEff: SNPs in the genome of Drosophila melanogaster strain w1118; iso-2; iso-3. Fly. (Austin).

[42] Schweiger R (2016). Fast and accurate construction of confidence intervals for heritability. Am. J. Hum. Genet..

[43] Price MN, Dehal PS, Arkin AP (2010). FastTree 2--approximately maximum-likelihood trees for large alignments. PLoS. One..

[44] Chewapreecha C (2014). Dense genomic sampling identifies highways of pneumococcal recombination. Nat. Genet..

[45] Cremers AJ (2015). The post-vaccine microevolution of invasive Streptococcus pneumoniae. Sci. Rep..

[46] Croucher NJ (2015). Rapid phylogenetic analysis of large samples of recombinant bacterial whole genome sequences using Gubbins. Nucleic Acids Res..

[47] Murrell B (2013). FUBAR: a fast, unconstrained bayesian approximation for inferring selection. Mol. Biol. Evol..

[48] Murrell B (2012). Detecting individual sites subject to episodic diversifying selection. PLoS. Genet..

[49] Pond SL, Frost SD, Muse SV (2005). HyPhy: hypothesis testing using phylogenies. Bioinformatics.

[50] Pozzi G (1996). Competence for genetic transformation in encapsulated strains of Streptococcus pneumoniae: two allelic variants of the peptide pheromone. J. Bacteriol..

[51] Regev-Yochay G, Trzcinski K, Thompson CM, Malley R, Lipsitch M (2006). Interference between Streptococcus pneumoniae and Staphylococcus aureus: In vitro hydrogen peroxide-mediated killing by Streptococcus pneumoniae. J. Bacteriol..

[52] Li Y, Thompson CM, Lipsitch M (2014). A modified Janus cassette (Sweet Janus) to improve allelic replacement efficiency by high-stringency negative selection in Streptococcus pneumoniae. PLoS. One..

[53] Brauner A, Fridman O, Gefen O, Balaban NQ (2016). Distinguishing between resistance, tolerance and persistence to antibiotic treatment. Nat. Rev. Microbiol..

[54] Fridman O, Goldberg A, Ronin I, Shoresh N, Balaban NQ (2014). Optimization of lag time underlies antibiotic tolerance in evolved bacterial populations. Nature.

[55] Team, R. C. R: A language and environment for statistical computing. (https://www.R-project.org, 2015).

