# Supplementary material for: Genome-wide association analyses of invasive pneumococcal isolates identify a missense bacterial mutation associated with meningitis
Source: Nat Commun. 2019 Jan 14;10:178. doi: 10.1038/s41467-018-07997-y (PMC6331587; doi:10.1038/s41467-018-07997-y)
Supplement: Supplementary file 1 — Supplementary Information [file 41467_2018_7997_MOESM1_ESM.pdf]

Supplementary Information

Genome-Wide Association Analyses of Invasive Pneumococcal Isolates Identify a Missense Bacterial Mutation Associated with Meningitis

Li et al

Supplementary Figure 1

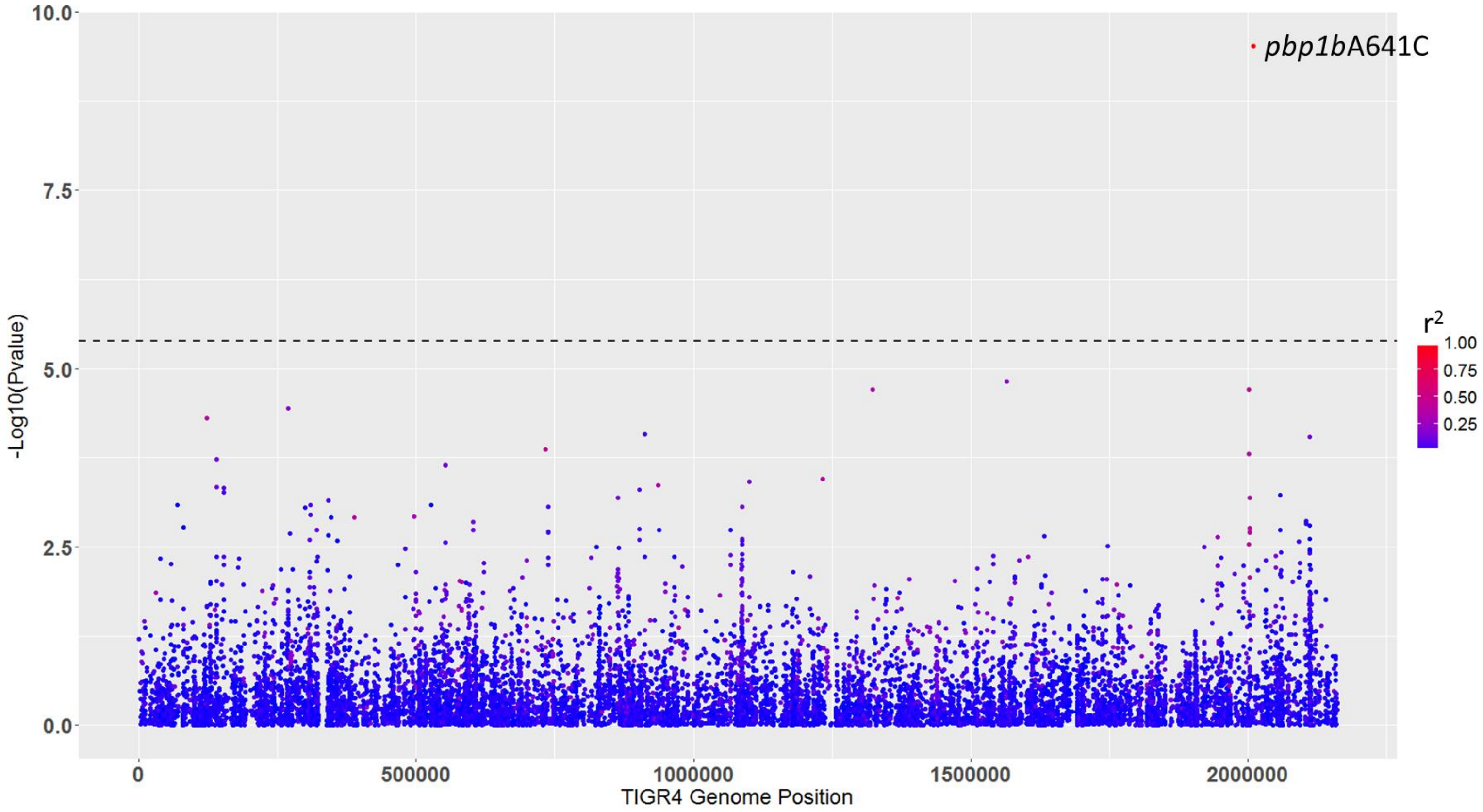

Supplementary Figure 1. Association between pneumococcal genome-wide non-synonymous SNPs and meningitis in the combined dataset (n=4572). P-values (-log10 transformed) of all 12,096 non-synonymous SNPs assessed by a linear mixed-effects model (LMM) controlling for population structure are shown. Dashed line indicates the threshold for Bonferroni corrected $p<0.05$. SNPs are colored according to their linkage disequilibrium (r^2 value) with the lead variant, *pbp1b*A641C (TIGR4 T2008526G).

Supplementary Figure 2

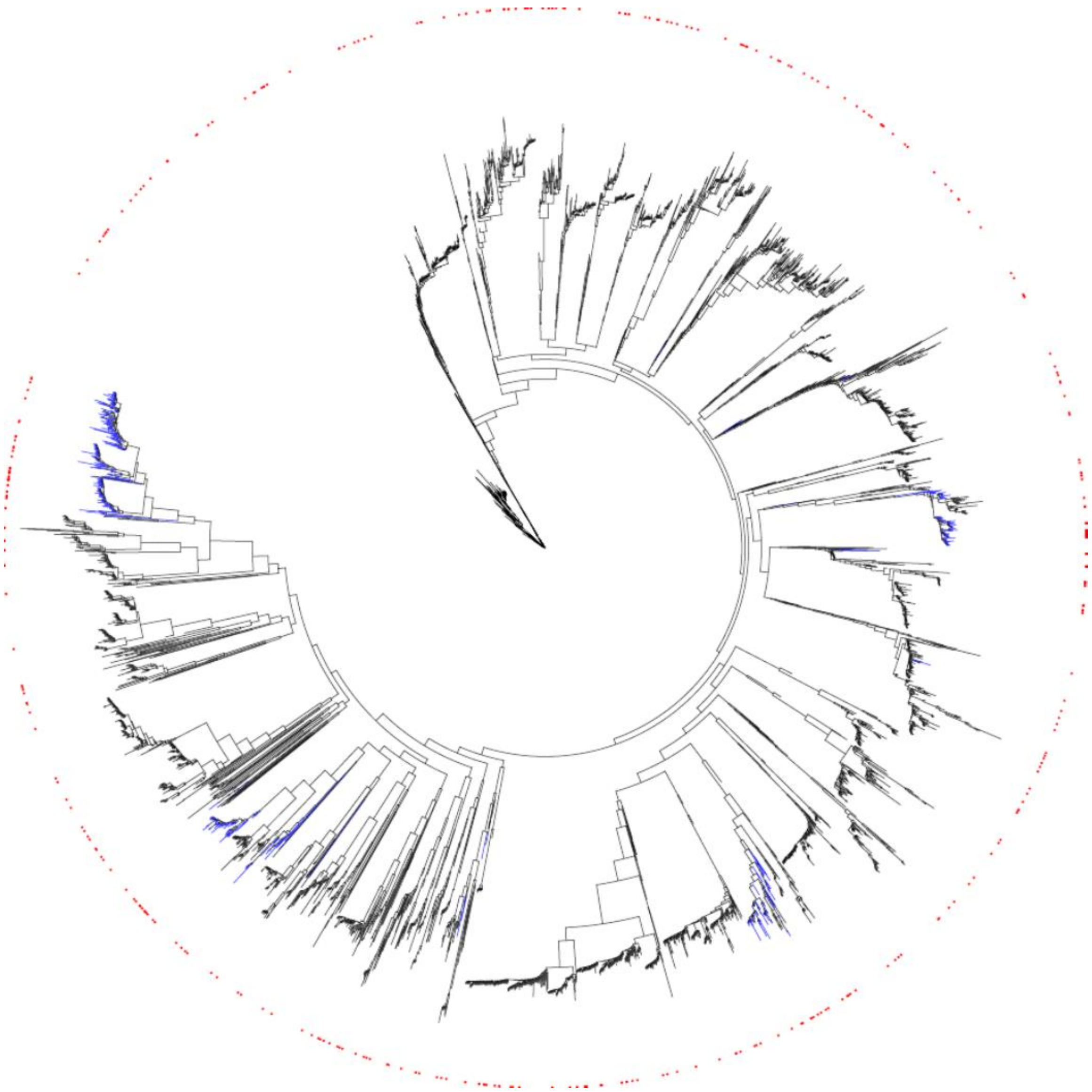

Supplementary Figure 2. A maximum-likelihood phylogenetic tree of the combined dataset isolates (n=4572) based on 57, 765 genome-wide SNPs. Blue branch tips indicate isolates with the *pbp1b*641C genotype. Red dots in the outside circle indicate isolates from meningitis patients.

Supplementary Figure 3

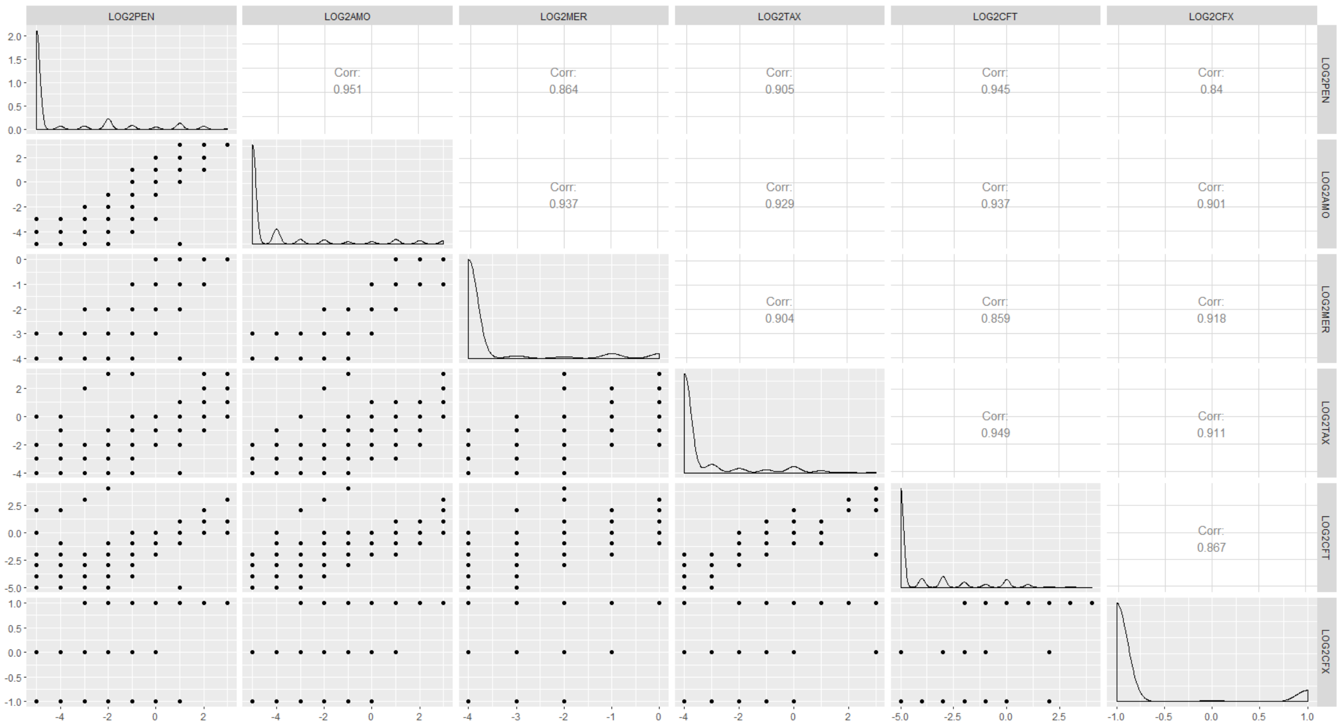

Supplementary Figure 3. Pair-wise scatter plot of log2 transformed MIC for six β-lactam antibiotics.

Supplementary Figure 4

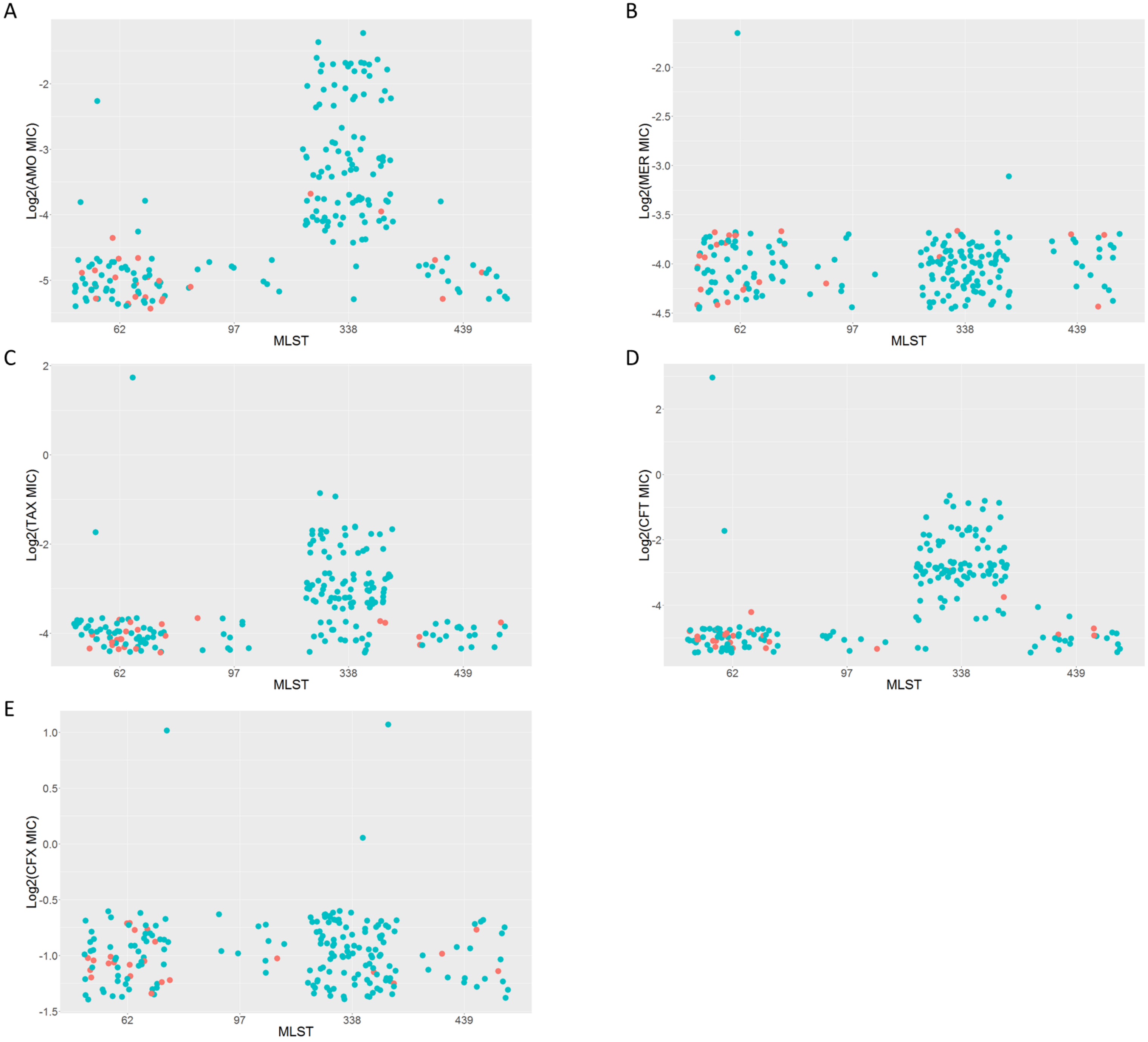

Supplementary Figure 4. Comparison of minimum inhibitory concentration (MIC) distribution between the non-*pbp1b*641C isolates (red) and *pbp1b*641C isolates (green) within the same multilocus sequence type (MLST). MIC (log2 transformed) distributions of five antibiotics are shown: Amoxicillin (A), Meropenem (B), Cefotaxime (C), Ceftriaxone (D), and Cefuroxime (E). Only the top four MLSTs containing both *pbp1b*641C and non-*pbp1b*641C isolates are included.

Supplementary Figure 5

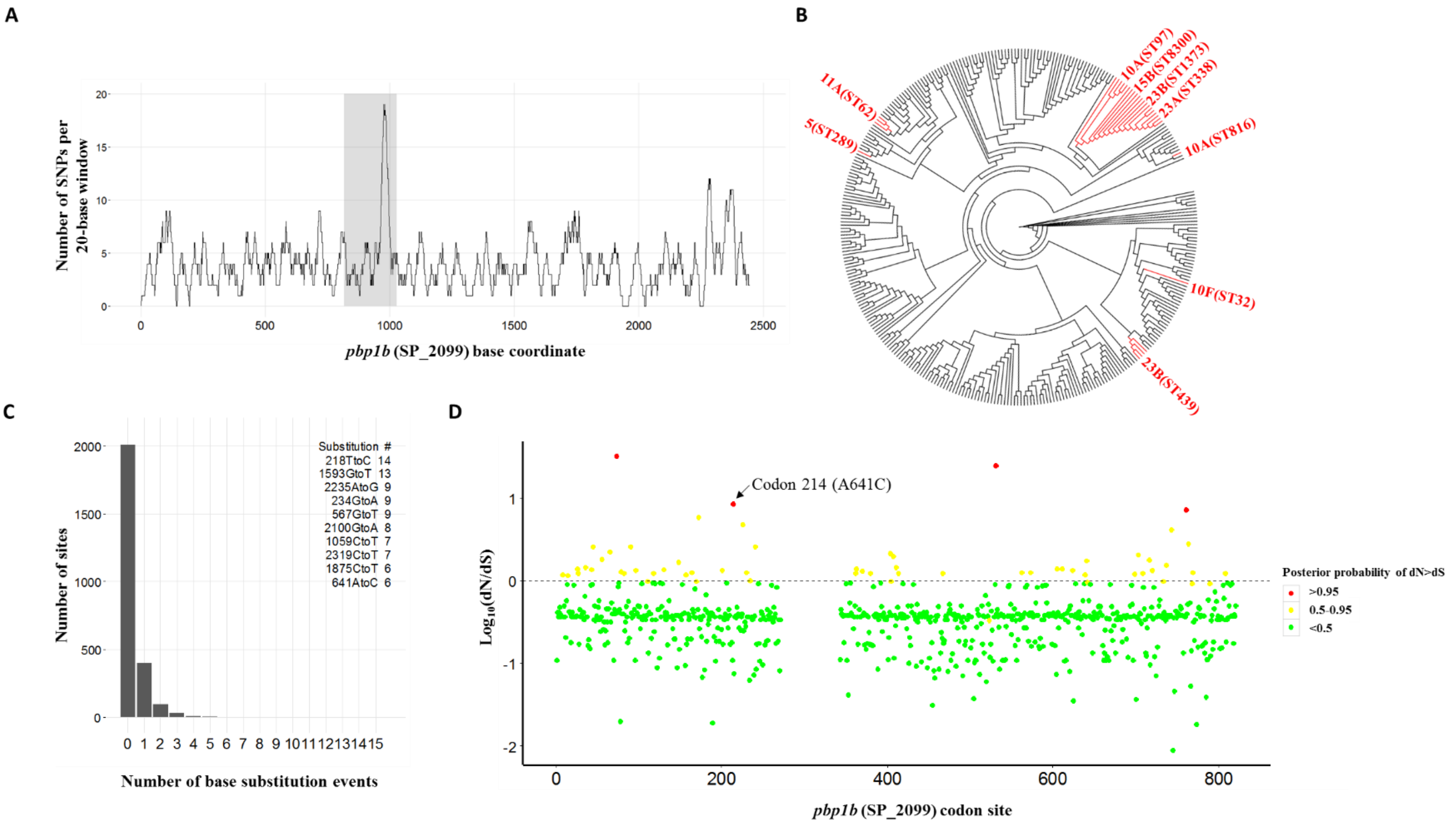

Supplementary Figure 5. Evidence of the *pbp1b*641 A to C substitution associating with selection advantage. (A) Density of single nucleotide polymorphism (SNP) within the *pbp1b* gene based on an alignment of 326 unique *pbp1b* sequences, of which 309 were from the two study cohorts and 17 were reference sequences. Number of SNPs within each 20-base sliding window is shown. Grey area indicates the region potentially affected by horizontal transfer, which was identified by the Gubbins software. (B) A cladogram of the 326 *pbp1b* gene sequences. Clades of *pbp1b* gene containing the 641C genotype are shown in read. Text labels indicate the representative serotype (MLST) found within each 641C-containing clade. (C) A histogram of the number of substitution event(s) at each base inferred from the phylogenetic analysis in (A). No substitution was observed for 2001 of the 2466 bases in the *pbp1b* reference sequence (SP_2099), while six independent A to C changes at position 641 were inferred, corresponding to the 6 red clades in (B). (C) Detection of site-specific pervasive diversifying positive selection using the partition alignment. The ratio between non-synonymous substitution rate (dN) and synonymous substitution rate (dS) was estimated by a fast, unconstrained Bayesian approximation method. The estimated dN/dS ratio ($\log_{10}$ transformed) is shown for each codon site. Dots are colored according to the posterior probabilities of dN/dS>1. Codon 273-343 were excluded from the analysis because they were within the region potentially affected by horizontal transfer.

Supplementary Figure 6

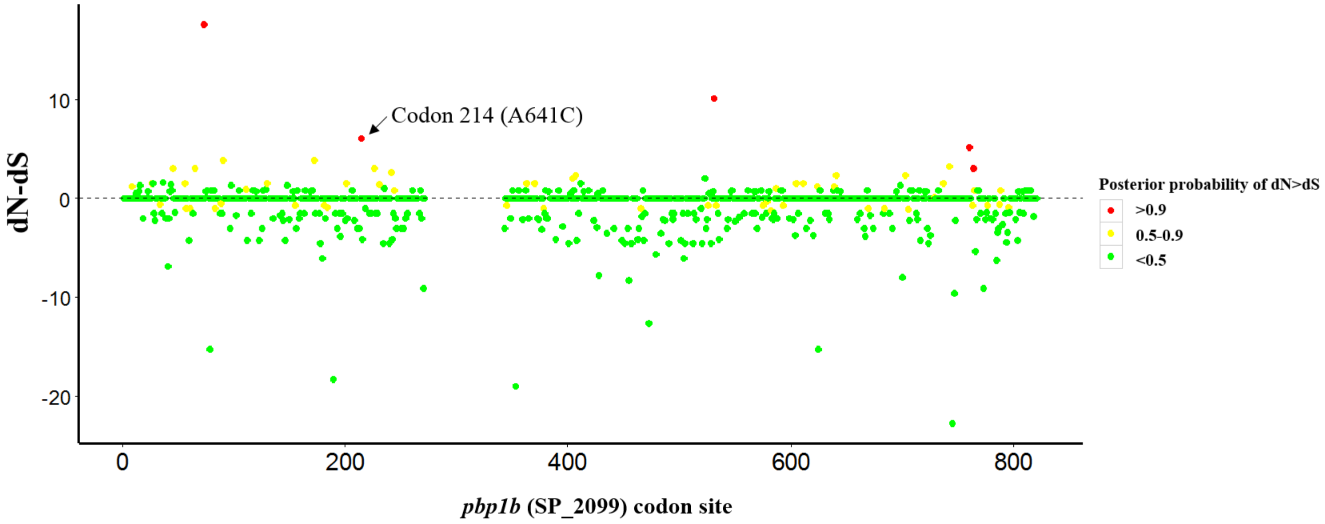

Supplementary Figure 6. Detection of site-specific diversifying positive selection using Mixed Effects Model of Evolution (MEME). The difference between non-synonymous substitution rate (dN) and synonymous substitution rate (dS) was estimated by the MEME method implemented in the HyPhy package. The estimated dN-dS is shown for each codon site. Dots are colored according to the posterior probabilities of dN>dS. Codon 273-343 were excluded from the analysis because they were within the region potentially affected by horizontal transfer.

Supplementary Tables

Supplementary Table 1. Association between candidate variant and increased Amoxicillin (AMO) MIC

Phenotype [a]	SNP [b]	Gene	AA change	Coefficient [c]	SE [d]	p-value
AMO MIC	T2008526G	pbp1b	N214T	-0.021	0.052	0.68
AMO MIC	A307891G	pbp2x	T338A	0.21	0.032	7.9×10^{-11}
AMO MIC	A308029G	pbp2x	R384G	0.14	0.030	8.4×10^{-7}
AMO MIC	C308515G	pbp2x	L546V	0.38	0.041	8.3×10^{-21}

a. Log_2 transformed MIC (μg ml^{-1}) of the indicated beat-lactam antibiotic
b. Relative to reference TIGR4 genome. First letter is the reference allele
c. Increase in log_2(MIC) associated with the alternative allele compared to the reference allele, after controlling for population structure
d. Standard error of the coefficient

Supplementary Table 2. Association between candidate variant and increased Meropenem (MER) MIC

Phenotype [a]	SNP [b]	Gene	AA change	Coefficient [c]	SE [d]	p-value
MER MIC	T2008526G	pbp1b	N214T	-0.028	0.027	0.30
MER MIC	A307891G	pbp2x	T338A	0.040	0.017	0.016
MER MIC	A308029G	pbp2x	R384G	0.045	0.015	0.0031
MER MIC	C308515G	pbp2x	L546V	0.15	0.022	1.1×10^{-11}

a. Log_2 transformed MIC ($\mu g\ ml^{-1}$) of the indicated beat-lactam antibiotic

b. Relative to reference TIGR4 genome. First letter is the reference allele

c. Increase in log_2(MIC) associated with the alternative allele compared to the reference allele, after controlling for population structure

d. Standard error of the coefficient

Supplementary Table 3. Association between candidate variant and increased Cefotaxime (TAX) MIC

Phenotype [a]	SNP [b]	Gene	AA change	Coefficient [c]	SE [d]	p-value
TAX MIC	T2008526G	pbp1b	N214T	-0.025	0.043	0.56
TAX MIC	A307891G	pbp2x	T338A	0.033	0.025	0.18
TAX MIC	A308029G	pbp2x	R384G	-0.0085	0.023	0.72
TAX MIC	C308515G	pbp2x	L546V	0.27	0.033	2.1×10^{-15}

a. Log_2 transformed MIC (µg ml^{-1}) of the indicated beat-lactam antibiotic
b. Relative to reference TIGR4 genome. First letter is the reference allele
c. Increase in log_2(MIC) associated with the alternative allele compared to the reference allele, after controlling for population structure
d. Standard error of the coefficient

Supplementary Table 4. Association between candidate variant and increased Ceftriaxone (CFT) MIC

Phenotype [a]	SNP [b]	Gene	AA change	Coefficient [c]	SE [d]	p-value
CFT MIC	T2008526G	pbp1b	N214T	-0.0018	0.053	0.97
CFT MIC	A307891G	pbp2x	T338A	0.14	0.031	8.1×10^{-6}
CFT MIC	A308029G	pbp2x	R384G	0.12	0.030	6.2×10^{-5}
CFT MIC	C308515G	pbp2x	L546V	0.40	0.041	2.8×10^{-22}

a. Log_2 transformed MIC (µg ml^{-1}) of the indicated beat-lactam antibiotic

b. Relative to reference TIGR4 genome. First letter is the reference allele

c. Increase in log_2(MIC) associated with the alternative allele compared to the reference allele, after controlling for population structure

d. Standard error of the coefficient

Supplementary Table 5. Association between candidate variant and increased Cefuroxime (CFX) MIC

Phenotype [a]	SNP [b]	Gene	AA change	Coefficient [c]	SE [d]	p-value
CFX MIC	T2008526G	pbp1b	N214T	-0.019	0.018	0.29
CFX MIC	A307891G	pbp2x	T338A	0.054	0.010	2.9×10^{-7}
CFX MIC	A308029G	pbp2x	R384G	0.030	0.0099	2.7×10^{-3}
CFX MIC	C308515G	pbp2x	L546V	0.13	0.014	2.1×10^{-21}

a. Log_2 transformed MIC (μg ml^{-1}) of the indicated beat-lactam antibiotic

b. Relative to reference TIGR4 genome. First letter is the reference allele

c. Increase in log_2(MIC) associated with the alternative allele compared to the reference allele, after controlling for population structure

d. Standard error of the coefficient

Supplementary Table 6. Candidate variant effect size estimation adjusting for patient age, pneumococcal serotype and Penicillin (PEN) susceptibility

Variable	Effects	Mixed-Effects logistic regression OR (95% CI) [a]	p-value
***pbp1b*641C**	Fixed		
No		Reference	NA
Yes		2.46 (1.49 to 4.06)	<0.001
**Patient Age (years)**	Fixed		
<2		1.98 (1.14 to 3.46)	0.016
2-4		0.33 (0.08 to 1.41)	0.135
5-17		1.37 (0.60 to 3.19)	0.458
18-64		Reference	NA
>64		0.46 (0.32 to 0.67)	<0.001
**PEN Susceptible**	Fixed		
Yes		Reference	
No		1.50 (0.98 to 2.29)	0.063
**Serotype**	Random		

a. Mixed-Effects logistic regression model with binary outcome (meningitis vs. non-meningitis) and the indicated explanatory variables.

Supplementary Table 7. Candidate variant effect size estimation adjusting for patient age, pneumococcal serotype and Amoxicillin (AMO) susceptibility

Variable	Effects	Mixed-Effects logistic regression OR (95% CI) [a]	p-value
***pbp1b*641C**	Fixed		
No		Reference	NA
Yes		2.95 (1.83 to 4.75)	<0.001
**Patient Age (years)**	Fixed		
<2		1.95 (1.11 to 3.40)	0.019
2-4		0.35 (0.08 to 1.49)	0.154
5-17		1.39 (0.61 to 3.18)	0.434
18-64		Reference	NA
>64		0.46 (0.32 to 0.67)	<0.001
**AMO Susceptible**	Fixed		
Yes		Reference	
No		2.59 (1.31 to 5.10)	0.006
**Serotype**	Random		

a. Mixed-Effects logistic regression model with binary outcome (meningitis vs. non-meningitis) and the indicated explanatory variables.

Supplementary Table 8. Candidate variant effect size estimation adjusting for patient age, pneumococcal serotype and Meropenem (MER) susceptibility

Variable	Effects	Mixed-Effects logistic regression OR (95% CI) [a]	p-value
***pbp1b*641C**	Fixed		
No		Reference	NA
Yes		3.00 (1.85 to 4.87)	<0.001
**Patient Age (years)**	Fixed		
<2		1.94 (1.11 to 3.39)	0.020
2-4		0.35 (0.08 to 1.48)	0.153
5-17		1.39 (0.61 to 3.19)	0.432
18-64		Reference	NA
>64		0.45 (0.31 to 0.66)	<0.001
**MER Susceptible**	Fixed		
Yes		Reference	
No		2.20 (1.18 to 4.07)	0.013
**Serotype**	Random		

a. Mixed-Effects logistic regression model with binary outcome (meningitis vs. non-meningitis) and the indicated explanatory variables.

Supplementary Table 9. Candidate variant effect size estimation adjusting for patient age, pneumococcal serotype and Cefotaxime (TAX) susceptibility

**Variable**	**Effects**	**Mixed-Effects logistic regression OR (95% CI)** [a]	**p-value**
***pbp1b*641C**	Fixed		
No		Reference	NA
Yes		2.96 (1.84 to 4.74)	<0.001
**Patient Age (years)**	Fixed		
<2		1.95 (1.12 to 3.40)	0.019
2-4		0.35 (0.08 to 1.52)	0.162
5-17		1.39 (0.61 to 3.18)	0.432
18-64		Reference	NA
>64		0.46 (0.32 to 0.67)	<0.001
**TAX Susceptible**	Fixed		
Yes		Reference	
No		2.10 (1.16 to 3.82)	0.015
**Serotype**	Random		

a. Mixed-Effects logistic regression model with binary outcome (meningitis vs. non-meningitis) and the indicated explanatory variables.

Supplementary Table 10. Candidate variant effect size estimation adjusting for patient age, pneumococcal serotype and Ceftriaxone (CFT) susceptibility

Variable	Effects	Mixed-Effects logistic regression OR (95% CI) [a]	p-value
***pbp1b*641C**	Fixed		
No		Reference	NA
Yes		2.99 (1.85 to 4.82)	<0.001
**Patient Age (years)**	Fixed		
<2		1.96 (1.12 to 3.42)	0.018
2-4		0.35 (0.08 to 1.49)	0.156
5-17		1.40 (0.61 to 3.19)	0.428
18-64		Reference	NA
>64		0.46 (0.31 to 0.66)	<0.001
**CFT Susceptible**	Fixed		
Yes			
No		2.16 (1.19 to 3.91)	0.011
**Serotype**	Random		

a. Mixed-Effects logistic regression model with binary outcome (meningitis vs. non-meningitis) and the indicated explanatory variables.

Supplementary Table 11. Candidate variant effect size estimation adjusting for patient age, pneumococcal serotype and Cefuroxime (CFX) susceptibility

Variable	Effects	Mixed-Effects logistic regression OR (95% CI) [a]	p-value
***pbp1b*641C**	Fixed		
No		Reference	NA
Yes		3.09 (1.90 to 5.03)	<0.001
**Patient Age (years)**	Fixed		
<2		1.99 (1.14 to 3.47)	0.016
2-4		0.35 (0.08 to 1.49)	0.155
5-17		1.42 (0.62 to 3.27)	0.401
18-64		Reference	NA
>64		0.45 (0.31 to 0.65)	<0.001
**CFX Susceptible**	Fixed		
Yes		Reference	
No		2.27 (1.36 to 3.08)	0.002
**Serotype**	Random		

b. Mixed-Effects logistic regression model with binary outcome (meningitis vs. non-meningitis) and the indicated explanatory variables.

Supplementary Table 12. Candidate variant effect size estimation adjusting for patient age, pneumococcal serotype and Penicillin (PEN) minimum inhibitory concentration (MIC)

Variable	Effects	Mixed-Effects logistic regression OR (95% CI) [a]	p-value
***pbp1b*641C**	Fixed		
No		Reference	NA
Yes		2.61 (1.58 to 4.51)	<0.001
**Patient Age (years)**	Fixed		
<2		1.95 (1.11 to 3.34)	0.019
2-4		0.33 (0.08 to 1.41)	0.136
5-17		1.39 (0.61 to 3.19)	0.434
18-64		Reference	NA
>64		0.45 (0.31 to 0.66)	<0.001
**Log2(PEN MIC)**	Fixed		
Continuous (range -5 to 3)		1.13 (1.03 to 1.24)	0.009
**Serotype**	Random		

a. Mixed-Effects logistic regression model with binary outcome (meningitis vs. non-meningitis) and the indicated explanatory variables.

Supplementary Table 13. Candidate variant effect size estimation adjusting for patient age, pneumococcal serotype and Amoxicillin (AMO) minimum inhibitory concentration (MIC)

Variable	Effects	Mixed-Effects logistic regression OR (95% CI) [a]	p-value
***pbp1b*641C**	Fixed		
No		Reference	NA
Yes		2.84 (1.72 to 4.69)	<0.001
**Patient Age (years)**	Fixed		
<2		1.91 (1.09 to 3.34)	0.023
2-4		0.34 (0.08 to 1.45)	0.145
5-17		1.39 (0.60 to 3.18)	0.441
18-64		Reference	NA
>64		0.45 (0.31 to 0.66)	<0.001
**Log2(AMO MIC)**	Fixed		
Continuous (range -5 to 3)		1.13 (1.03 to 1.24)	0.006
**Serotype**	Random		

a. Mixed-Effects logistic regression model with binary outcome (meningitis vs. non-meningitis) and the indicated explanatory variables.

Supplementary Table 14. Candidate variant effect size estimation adjusting for patient age, pneumococcal serotype and Meropenem (MER) minimum inhibitory concentration (MIC)

Variable	Effects	Mixed-Effects logistic regression OR (95% CI) [a]	p-value
***pbp1b*641C**	Fixed		
No		Reference	NA
Yes		3.05 (1.88 to 4.97)	<0.001
**Patient Age (years)**	Fixed		
<2		1.93 (1.10 to 3.37)	0.021
2-4		0.35 (0.08 to 1.49)	0.154
5-17		1.41 (0.62 to 3.32)	0.418
18-64		Reference	NA
>64		0.45 (0.31 to 0.66)	<0.001
**Log2(MER MIC)**	Fixed		
Continuous (range -4 to 0)		1.27 (1.07 to 1.50)	0.006
**Serotype**	Random		

b. Mixed-Effects logistic regression model with binary outcome (meningitis vs. non-meningitis) and the indicated explanatory variables.

Supplementary Table 15. Candidate variant effect size estimation adjusting for patient age, pneumococcal serotype and Cefotaxime (TAX) minimum inhibitory concentration (MIC)

Variable	Effects	Mixed-Effects logistic regression OR (95% CI) [a]	p-value
***pbp1b*641C**	Fixed		
No		Reference	NA
Yes		2.83 (1.74 to 4.61)	<0.001
**Patient Age (years)**	Fixed		
<2		1.95 (1.12 to 3.41)	0.019
2-4		0.35 (0.08 to 1.50)	0.157
5-17		1.39 (0.61 to 3.20)	0.433
18-64		Reference	NA
>64		0.45 (0.31 to 0.66)	<0.001
**Log2(TAX MIC)**	Fixed		
Continuous (range -4 to 3)		1.19 (1.05 to 1.36)	0.006
**Serotype**	Random		

a. Mixed-Effects logistic regression model with binary outcome (meningitis vs. non-meningitis) and the indicated explanatory variables.

Supplementary Table 16. Candidate variant effect size estimation adjusting for patient age, pneumococcal serotype and Cefotriaxone (CFT) minimum inhibitory concentration (MIC)

Variable	Effects	Mixed-Effects logistic regression OR (95% CI) [a]	p-value
***pbp1b*641C**	Fixed		
No		Reference	NA
Yes		2.71 (1.66 to 4.43)	<0.001
**Patient Age (years)**	Fixed		
<2		1.96 (1.12 to 3.42)	0.019
2-4		0.34 (0.08 to 1.46)	0.157
5-17		1.39 (0.61 to 3.19)	0.433
18-64		Reference	NA
>64		0.45 (0.31 to 0.66)	<0.001
**Log2(CFT MIC)**	Fixed		
Continuous (range -5 to 4)		1.14 (1.03 to 1.26)	0.014
**Serotype**	Random		

a. Mixed-Effects logistic regression model with binary outcome (meningitis vs. non-meningitis) and the indicated explanatory variables.

Supplementary Table 17. Candidate variant effect size estimation adjusting for patient age, pneumococcal serotype and Cefuroxime (CFX) minimum inhibitory concentration (MIC)

Variable	Effects	Mixed-Effects logistic regression OR (95% CI) [a]	p-value
***pbp1b*641C**	Fixed		
No		Reference	NA
Yes		3.10 (1.66 to 4.43)	<0.001
**Patient Age (years)**	Fixed		
<2		1.97 (1.13 to 3.45)	0.019
2-4		0.35 (0.08 to 1.47)	0.157
5-17		1.42 (0.62 to 3.25)	0.433
18-64		Reference	NA
>64		0.45 (0.31 to 0.65)	<0.001
**Log2(CFX MIC)**	Fixed		
Continuous (range -1 to 1)		1.55 (1.17 to 2.04)	0.002
**Serotype**	Random		

a. Mixed-Effects logistic regression model with binary outcome (meningitis vs. non-meningitis) and the indicated explanatory variables.

Supplementary Table 18. Top five variants associated with meningitis identified by fitting linear mixed-effects model using the GEMMA software, exploratory sample

**Rank**	**Type**	**Gene Affected**	**p-value**
1	AAV	Penicillin-binding protein 1b (*pbp1b*)	3.85×10^{-6}
2	AAV	penicillin-binding protein 1A (*pbp1A*)	1.81×10^{-4}
3	AAV	ABC transporter, ATP-binding protein (SP670_913)	2.18×10^{-4}
4	AAV	Glycosyltransferase (SP670_913)	2.20×10^{-4}
5	AAV	penicillin-binding protein 1A (*pbp1A*)	3.42×10^{-4}

Supplementary Table 19. Top five variants associated with meningitis identified by fitting linear mixed-effects model using the GEMMA software, confirmatory cohort

**Rank**	**Type**	**Gene Affected**	**p-value**
1	AAV	Penicillin-binding protein 1b (*pbp1b*)	6.44×10^{-6}
2	GAP	primase 1 family protein (PAR136_1574)	5.12×10^{-6}
3	AAV	conserved hypothetical protein(SP670_1752)	1.29×10^{-5}
4	AAV	mannose-6-phosphate isomerase (MYY_0772)	2.13×10^{-5}
5	GAP	hypothetical protein (SPAR94_1544)	2.41×10^{-4}

Supplementary Table 20. Comparison of the exploratory sample isolates with the overall ABCs data

	Exploratory sample (n=2054)	ABCs Isolates [a] (n=56771)	Overall ABCs [b] (n=64126)
**Meningitis**			
No	1915 (93%)[c]	53348 (94%)	60341 (94%)
Yes	139 (7%)	3423 (6%)	3785 (6%)
**Patient age (years)**			
<2	848 (41%)	5394 (10%)	6265 (10%)
2-4	556 (27%)	2449 (4%)	2844 (4%)
5-17	125 (6%)	1940 (3%)	2185 (3%)
18-64	310 (15%)	28446 (50%)	31945 (50%)
>64	215 (10%)	18536 (33%)	20879 (33%)
Unknown	0 (0 %)	6 (0%)	8 (0%)
**Serotype Group**			
Non-PCV13[d]	835 (41%)	25978 (46%)	NA
PCV7[e]	565 (27%)	13699 (24%)	NA
PCV13 minus PCV7	654 (32%)	17019 (30%)	NA
Unknown	0 (0%)	75 (0%)	NA
**PEN MIC (μg/mL)**			
≤0.06	1373 (67%)	43044 (76%)	NA
≥0.12	681 (33%)	13710 (24%)	NA
Unknown	0 (0%)	17 (0%)	NA

a. ABCs data 1998-2014 where isolate available with MIC results
b. All ABCs data 1998-2014 (including cases without an isolate available)
c. Data are number (%). Some percentages do not total 100 because of rounding.
d. PCV13 serotypes are 1, 3, 4, 5, 6A, 6B, 7F, 9V, 14, 18C, 19A, 19F, and 23F.
e. PCV7 serotypes are 4, 6B, 9V, 14, 18C, 19F, and 23F.

Supplementary Table 21. Interpretive Standard for β-lactam antibiotics

Antibiotics	Breakpoint MIC (μg/mL)	
	Susceptible (S)	Non-Susceptible (NS)
Penicillin (PEN)	≤0.06	≥0.12
Amoxicillin (AMO)	≤2	≥4
Meropenem (MER)	≤0.25	≥0.5
Cefotaxime (TAX)	≤0.5	≥1
Ceftriaxone (CFT)	≤0.5	≥1
Cefuroxime (CFX)	≤0.5	≥1

Supplementary Table 22. List of the 20 *S. pneumoniae* complete genome sequences

Strain Name	Accession Number	URL	CDS prefix
TIGR4	NC_003028	https://www.ncbi.nlm.nih.gov/nuccore/NC_003028	SP
R6	NC_003098	https://www.ncbi.nlm.nih.gov/nuccore/NC_003098	spr
D39	NC_008533	https://www.ncbi.nlm.nih.gov/nuccore/NC_008533	SPD
Hungary19A	NC_010380	https://www.ncbi.nlm.nih.gov/nuccore/NC_010380	SPH
CGSP14	NC_010582	https://www.ncbi.nlm.nih.gov/nuccore/NC_010582	SPCG
G54	NC_011072	https://www.ncbi.nlm.nih.gov/nuccore/NC_011072	SPG
ATCC_700669	NC_011900	https://www.ncbi.nlm.nih.gov/nuccore/NC_011900	SPN23F
JJA	NC_012466	https://www.ncbi.nlm.nih.gov/nuccore/NC_012466	SPJ
P1031	NC_012467	https://www.ncbi.nlm.nih.gov/nuccore/NC_012467	SPP
70585	NC_012468	https://www.ncbi.nlm.nih.gov/nuccore/NC_012468	SP70585
Taiwan19F_14	NC_012469	https://www.ncbi.nlm.nih.gov/nuccore/NC_012469	SPT
TCH8431_19A	NC_014251	https://www.ncbi.nlm.nih.gov/nuccore/NC_014251	HMPREF0837
AP200	NC_014494	https://www.ncbi.nlm.nih.gov/nuccore/NC_014494	SPAP
670_6B	NC_014498]	https://www.ncbi.nlm.nih.gov/nuccore/NC_014498]	SP670
INV104	NC_017591	https://www.ncbi.nlm.nih.gov/nuccore/NC_017591	INV104
OXC141	NC_017592	https://www.ncbi.nlm.nih.gov/nuccore/NC_017592	SPNOXC
INV200	NC_017593	https://www.ncbi.nlm.nih.gov/nuccore/NC_017593	SPNINV200
ST556	NC_017769	https://www.ncbi.nlm.nih.gov/nuccore/NC_017769	MYY
gamPNI0373	NC_018630	https://www.ncbi.nlm.nih.gov/nuccore/NC_018630	SPNA45
SPNA54	NC_018594	https://www.ncbi.nlm.nih.gov/nuccore/NC_018594	HMPREF1038

Supplementary Table 23. Allele sequences used in SRST2 for *pbp1b*641C sequence typing

Allele	Sequence for typing
*pbp1b*641A	ATTCGTGCGACCTTGGGGAAATTTGTAGGTTTGGGTTCCTCTAGTGGGGGTTCAACCTTGACCCAGCAACTAATTA<br>AACAGCAGGTGGTTGGGGATGCGCCGACCTTGGCTCGTAAGGCGGCAGAGATTGTGGATGCTCTTGCCTTGGAA<br>CGCGCCATGAATAAAGATGAGATTTTAACGACCTATCTCAATGTGGCTCCCTTTGGCCGAAATAATAAGGGACAGA<br>ATATTGCAGGGGCTCGGCAAGCAGCTGAGGGAATTTTCGGTGTAGATGCCAGTCAGTTGACTGTTCCTCAAGCA
*pbp1b*641C	ATTCGTGCGACCTTGGGGAAATTTGTAGGTTTGGGTTCCTCTAGTGGGGGTTCAACCTTGACCCAGCAACTAATTA<br>AACAGCAGGTGGTTGGGGATGCGCCGACCTTGGCTCGTAAGGCGGCAGAGATTGTGGATGCTCTTGCCTTGGAA<br>CGCGCCATGACTAAAGATGAGATTTTAACGACCTATCTCAATGTGGCTCCCTTTGGCCGAAATAATAAGGGACAGA<br>ATATTGCAGGGGCTCGGCAAGCAGCTGAGGGAATTTTCGGTGTAGATGCCAGTCAGTTGACTGTTCCTCAAGCA

Supplementary Table 24. Laboratory strains and PCR primers used in this study

Strain or Primer	Description	Source or reference
**Strains**		
R6	A streptomycin-resistant R6 strain	J Bacteriol. 2006 Jul;188(13):4996-5001.(PMID:16788209)
SPNYL001	A TIGR4 strain derivative with the *cps* locus replaced by the Sweet-Janus cassette	PLoS One. 2014 Jun 24;9(6):e100510.(PMID: 24959661)
R6_SJ	R6 but *pbp1b*::*sacB-kan-rpsL*$^{+}$	This study
R6_641C	R6 but *pbp1b*:: *pbp1bA641C*	This study
**Primers**		
YL8001	GGCAAATCAAGATACTCCAAATATC	This study
YL8002	CGATCCTTAAGATTCAGCAAGCTTCCCC	This study
YL8003	TTGCTGAATCTTAAGGATCGATCCGTTTG	This study
YL8004	AGCAAAAACATTATGCTTTTGGACGTTTAG	This study
YL8005	AAAAGCATAATGTTTTTGCTGGAAAAATTCC	This study
YL8006	CTTCACGAGCCAAGACTTTTTG	This study
YL8007	GCCAAAATCATCCCCCACCC	This study
YL8008	CAATCTCATCAAGTGACAAGAAAGT	This study
YL8009	ATCTTTAGTCATGGCGCGTTCCAAG	This study
YL8010	GCCATGACTAAAGATGAGATTTTAACG	This study

Supplementary Table 25. Minimum inhibitory concentration (MIC) for the R6 and R6_641C strains measured by broth microdilution method

**Antibiotic**	**MIC (μg ml^{-1})**	
	**R6**	**R6_641C**
Ampicillin	=0.06	=0.06
Cefoxitin	=4	=4
Ceftaroline	<=0.12	<=0.12
Ceftizoxime	<=0.12	<=0.12
Ceftriaxone	<=0.06	<=0.06
Chloramphenicol	<=2	<=2
Clindamycin	<=0.06	<=0.06
Daptomycin	<=0.5	<=0.5
Doxycycline	=0.5	=0.25
Erythromycin	<=0.06	<=0.06
Levofloxacin	<=0.5	=1
Linezolid	<=1	<=1
Meropenem	<=0.06	<=0.06
Penicillin	<=0.03	<=0.03
Rifampin	<=0.5	<=0.5
Synercid	<=0.5	<=0.5
Trimeth-sulfa	<=0.12/2.38	<=0.12/2.38
Vancomycin	<=0.25	<=0.25
