# Supplementary material for: Genome-wide association analyses of invasive pneumococcal isolates identify a missense bacterial mutation associated with meningitis
Source: Nat Commun. 2019 Jan 14;10:178. doi: 10.1038/s41467-018-07997-y (PMC6331587; doi:10.1038/s41467-018-07997-y)
Supplement: Supplementary file 2 — Description of Additional Supplementary Files [file 41467_2018_7997_MOESM2_ESM.pdf]

Description of Additional Supplementary Files

File Name: Supplementary Data 1
Description: List of 2054 exploratory sample isolates.

File Name: Supplementary Data 2
Description: List of 2518 confirmatory cohort isolates.
